# Supplementary material for: Zebrafish capable of generating future state prediction error show improved active avoidance behavior in virtual reality
Source: Nat Commun. 2021 Sep 29;12:5712. doi: 10.1038/s41467-021-26010-7 (PMC8481257; doi:10.1038/s41467-021-26010-7)
Supplement: Supplementary file 1 — Supplementary information. [file 41467_2021_26010_MOESM1_ESM.pdf]

Supplementary Fig. 1

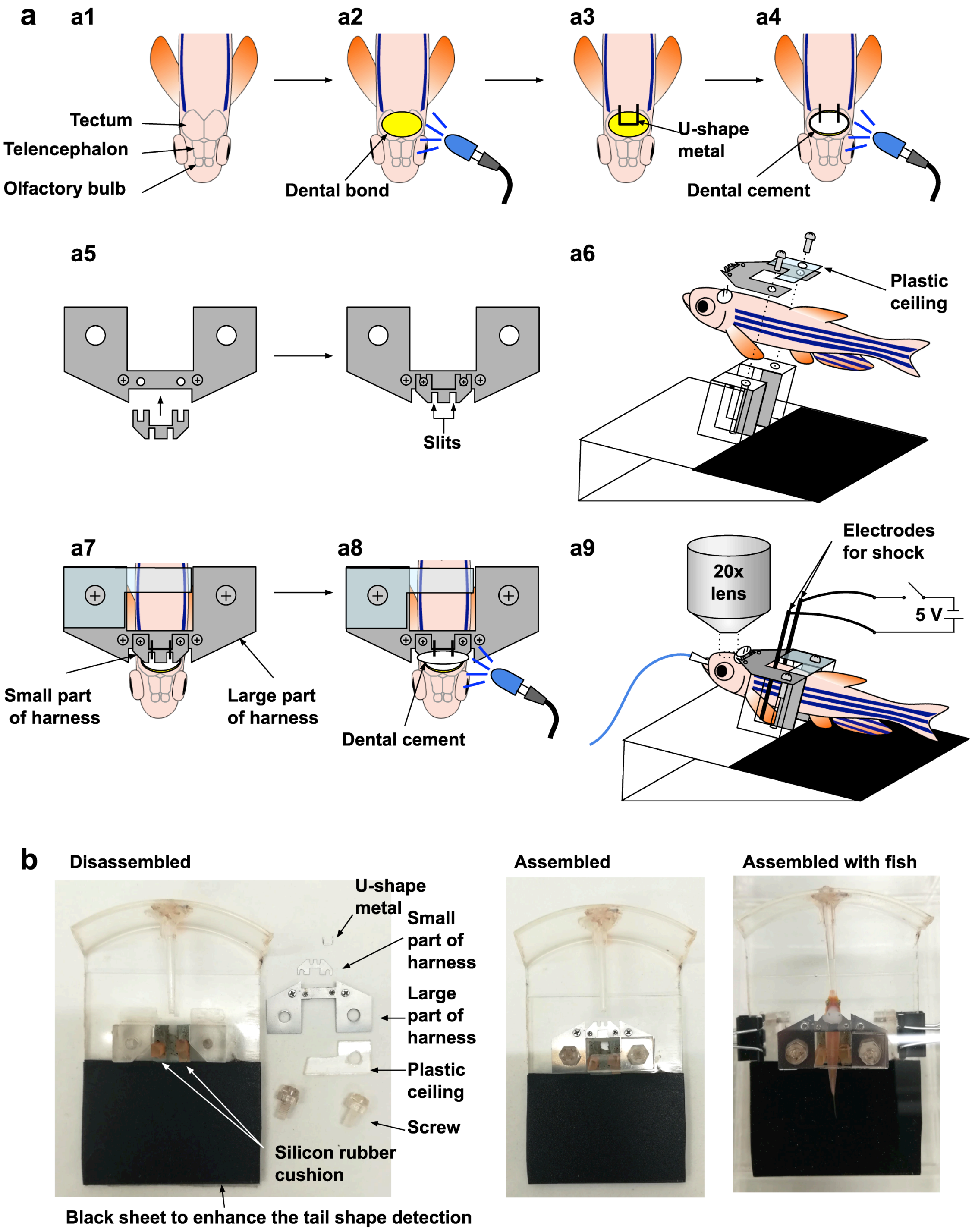

Supplementary Fig. 1. Fixation of live adult zebrafish

(**a**) Fixation of live adult zebrafish using a custom-made harness.

(a1) The skin was removed above the telencephalon and the tectum using micro knives. The skull was kept intact. (a2) After drying the surface, the dental bond (yellow) was pasted using a toothpick and illuminated with blue LED light for 10 s. (a3) The u-shaped metal was placed on the skull. (a4) The dental cement (white) was wrapped around the u-shaped metal using a toothpick and illuminated with blue LED light for 10 s. (a5) The harness was assembled for fixation. (a6) The fish was placed on the base of the fixation apparatus and the harness was fixed by two screws to the base and plastic ceiling. (a7) The tips of u-shaped metal were inserted into the slits of the assembled harness. (a8) Dental cement was added to the contact point between the u-shaped metal and the slits of the harness and illuminated with blue LED light for 10 s. (a9) The two needle electrodes used to deliver electric shocks were then placed.

(**b**) Images of the custom-made harness. Left panel, disassembled; center panel, assembled; right panel, assembled with fish.

Supplementary Fig. 2

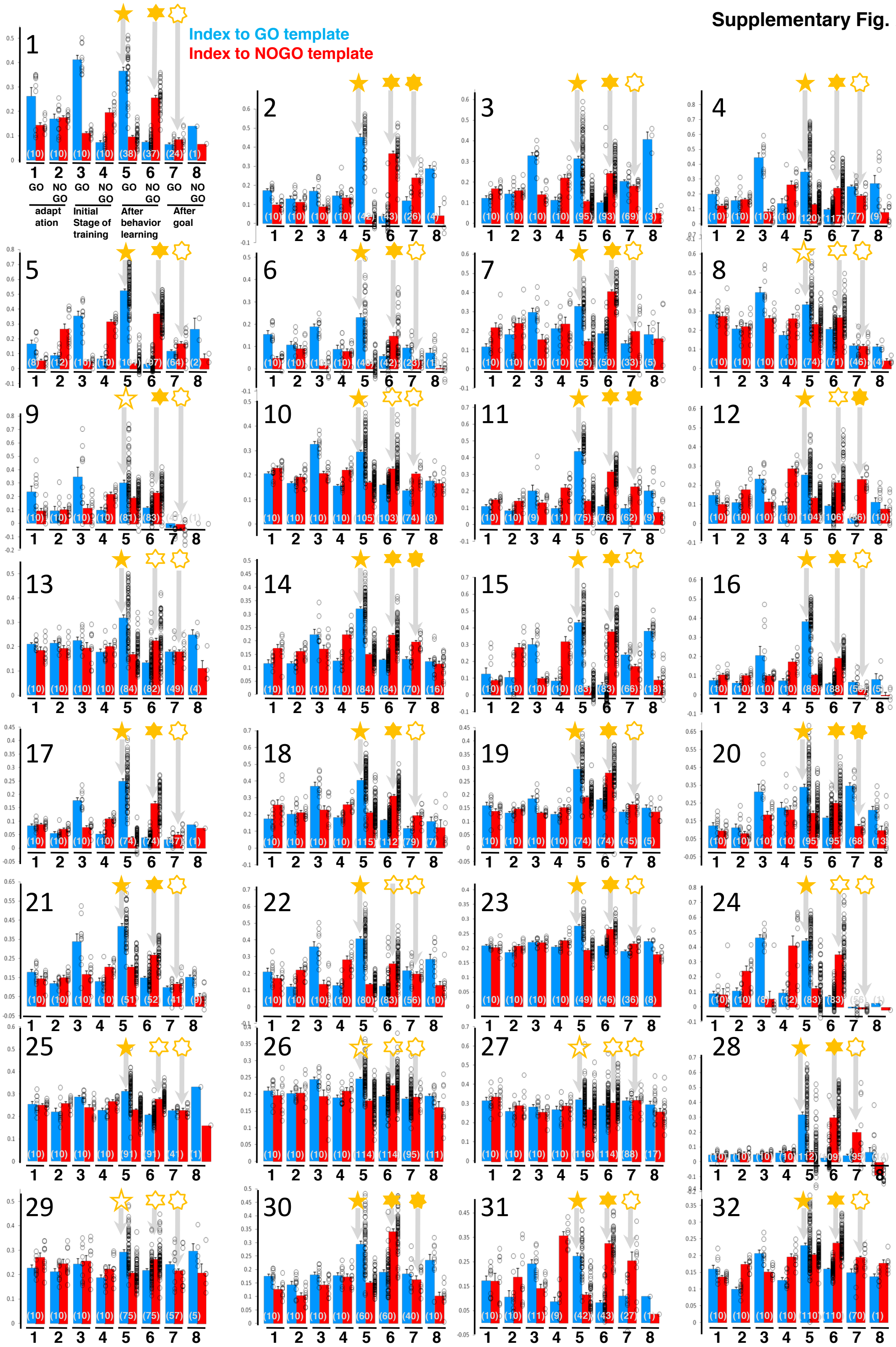

Supplementary Fig. 2. Results of template matching in all learner fish

Each graph illustrates the result of an individual fish. The cyan and red columns indicate the mean calculated value using GO and NOGO templates, respectively. The numbers 1-8 indicate the stage of behavioral learning and task category. 1, GO trials in the adaptation period; 2, NOGO trials in the adaptation period; 3, GO trials in the initial stage of training; 4, NOGO trials in the initial stage of training; 5, GO trials after behavioral learning was established; 6, NOGO trials after behavioral learning was established; 7, after goal in successful GO trials after reaching the color change border; 8, after goal in failed NOGO trials after reaching the color change border. Circles, data from each trial. Pentagonal star, higher index to GO template in GO trials after behavioral learning than that in GO trials in the adaptation period; hexagonal star, higher index to NOGO template in NOGO trials after behavioral learning than that in NOGO trials in the adaptation period; heptagonal star, higher index to NOGO template after goal in GO trials after behavioral learning than that in NOGO trials in the adaptation period; open stars, no significant difference in the index to the indicated template between indicated periods. Error Bars indicate the SEM. The numbers in parentheses are the number of trials used in the statistics.

Supplementary Fig. 3

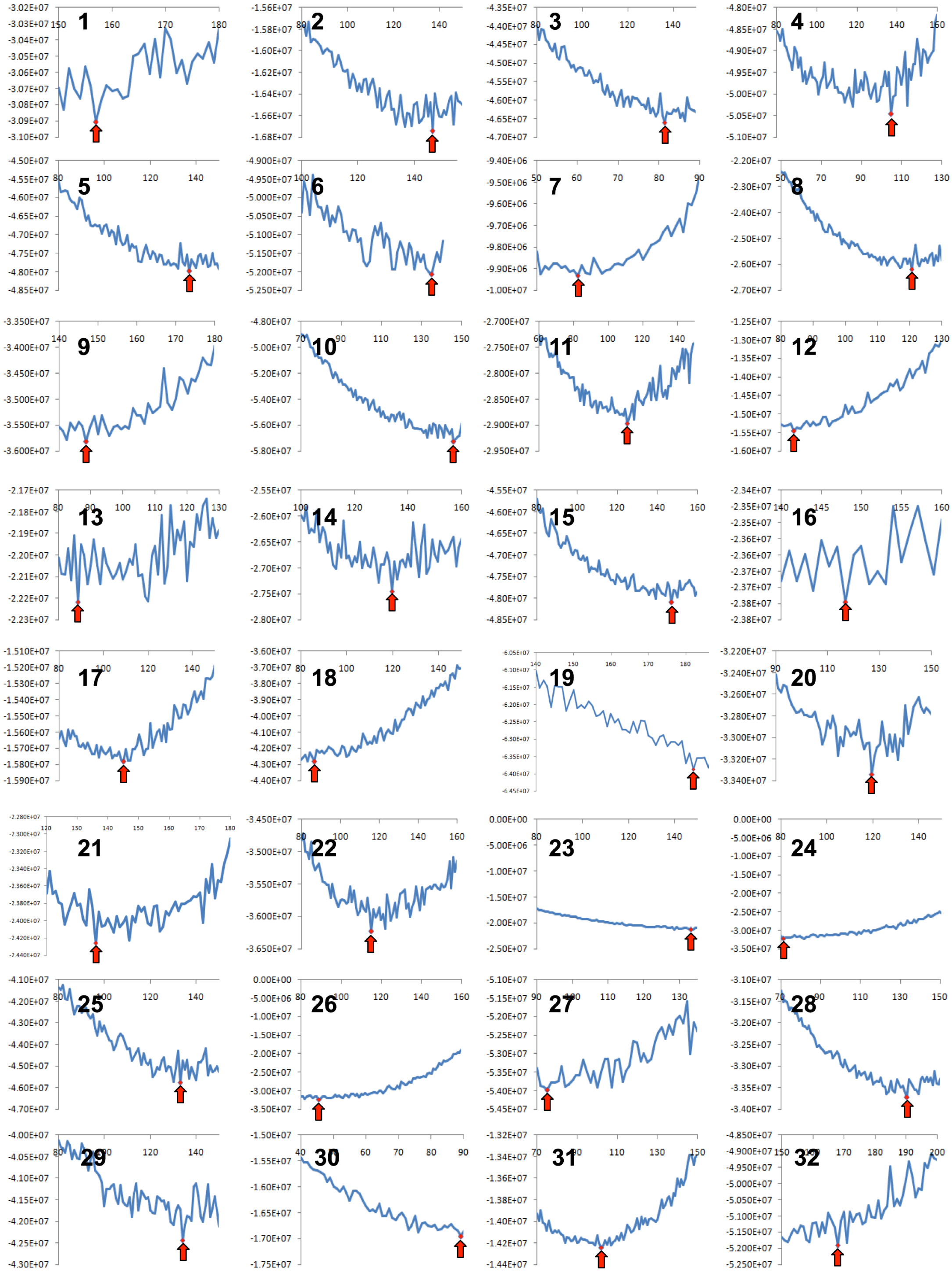

Supplementary Fig. 3. Akaike Information Criteria (AIC) curves in all learner fish.
Each graph shows the AIC curve of each learner fish for determination of the number of patterns. x axis, component number; y axis, AIC value. Red arrow and dot in each graph indicate the minimum value of AIC.

Supplementary Fig. 4

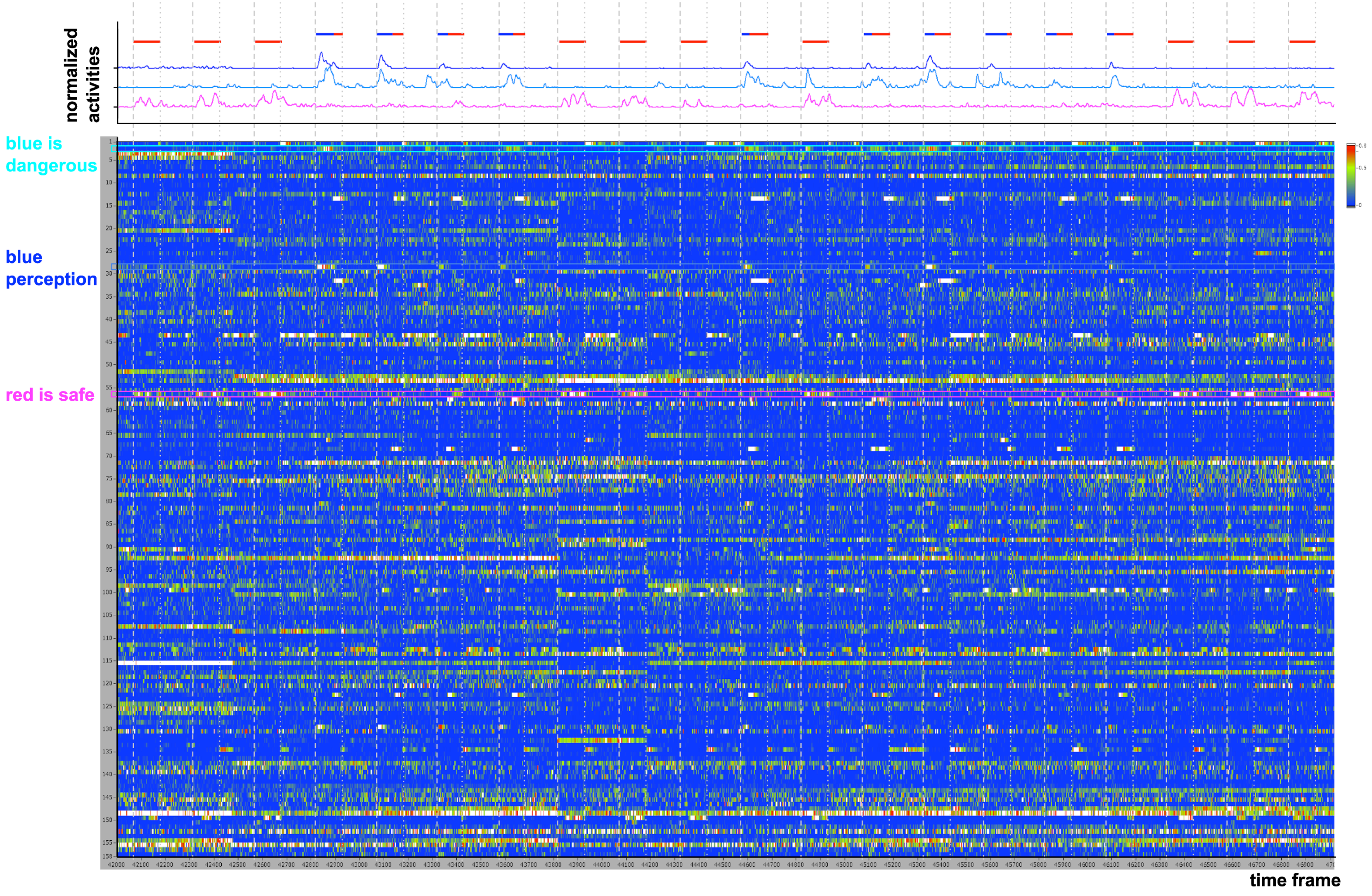

Supplementary Fig. 4. Time-lapse activity changes of all ensembles of fish 1 after behavioral learning.

Top: Activity of the neural ensembles encoding blue perception (blue line), blue is dangerous (cyan line) and red is safe (magenta line) normalized by the self-maximum value of fish 1. Notation is the same as in Fig. 3b.

Bottom: Time-lapse activity changes of all ensembles of fish 1. Colored boxes indicate the identified ensembles in accordance with the coloring in the top panel, *i.e.* blue box, the ensembles encoding blue perception; cyan box, the ensemble encoding that blue is dangerous; magenta box, the ensemble encoding that red is safe.

Dashed line, the onset of trial. Dotted line, the end of trial.

Supplementary Fig. 5

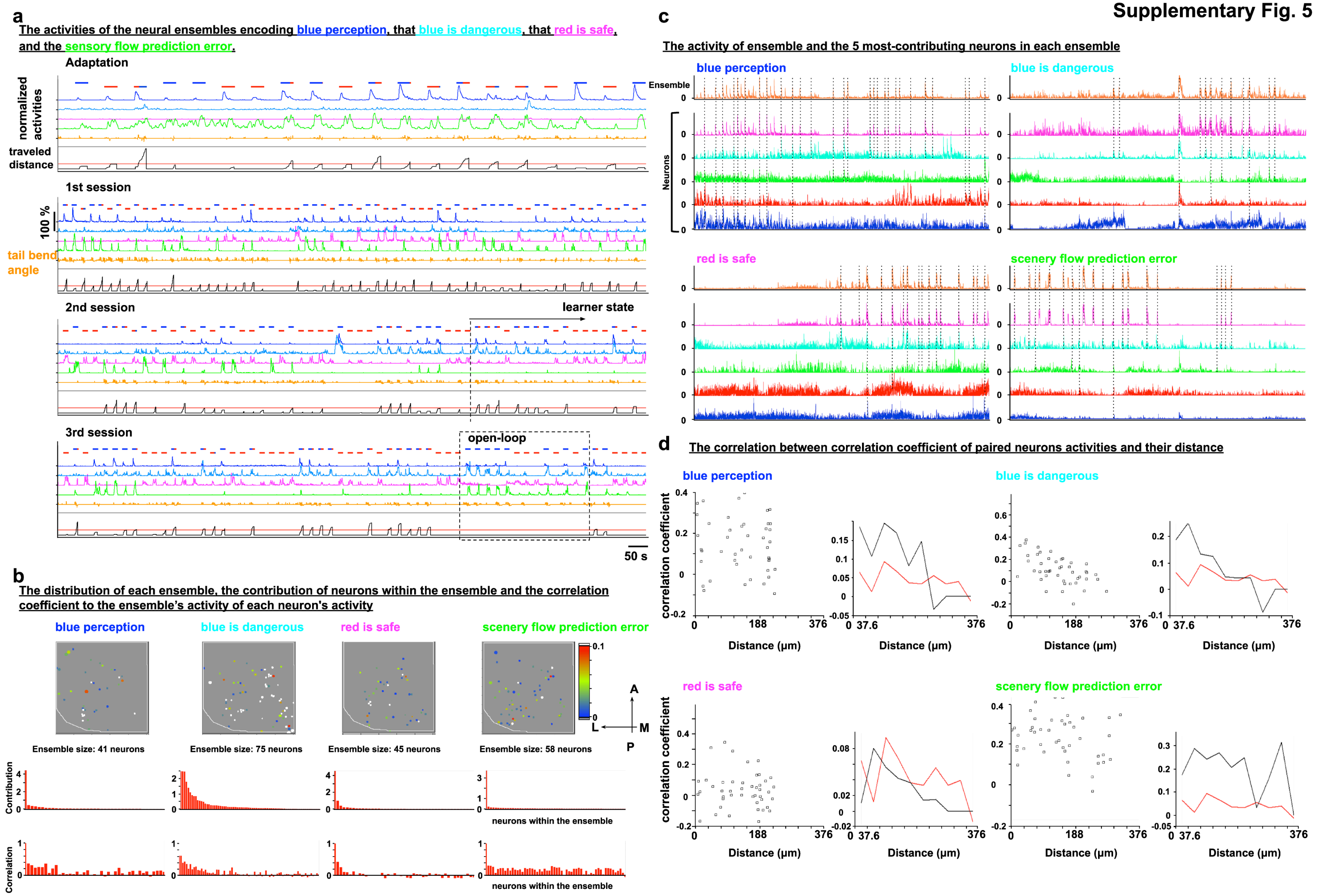

Supplementary Fig. 5. Data of Fish 1

(a) Activity of the identified ensembles. Notation is the same as in Fig. 3b. Blue line, activity of the ensemble encoding blue perception; cyan line, activity of the ensemble encoding that blue is dangerous; magenta line, activity of the ensemble encoding that red is safe; green line, activity of the ensemble encoding the scenery flow prediction error.

(**b**) Spatial distribution of neurons contributing to each ensemble. The color indicates the contribution of each neuron to the ensemble (top panels). A, anterior direction; P, posterior direction; L, lateral direction; M, medial direction. The contribution of neurons within each ensemble (middle panels). The correlation coefficient of activity of neurons within each ensemble and entire ensemble's activity (bottom panels).

(**c**) Activity of the ensemble (top trace) and the five most-contributing neurons in the ensemble (descending order from the top). Dotted lines indicate the timing when the neurons showed simultaneous activation with the ensemble.

(**d**) Relationship between the correlation coefficient and distance among the 10 most-contributing neurons in the ensembles. Black line denotes the averaged correlation in this fish. Red line denotes the average of 10 shuffled data (see Methods). The positively correlated neurons tended to accumulate at close distances.

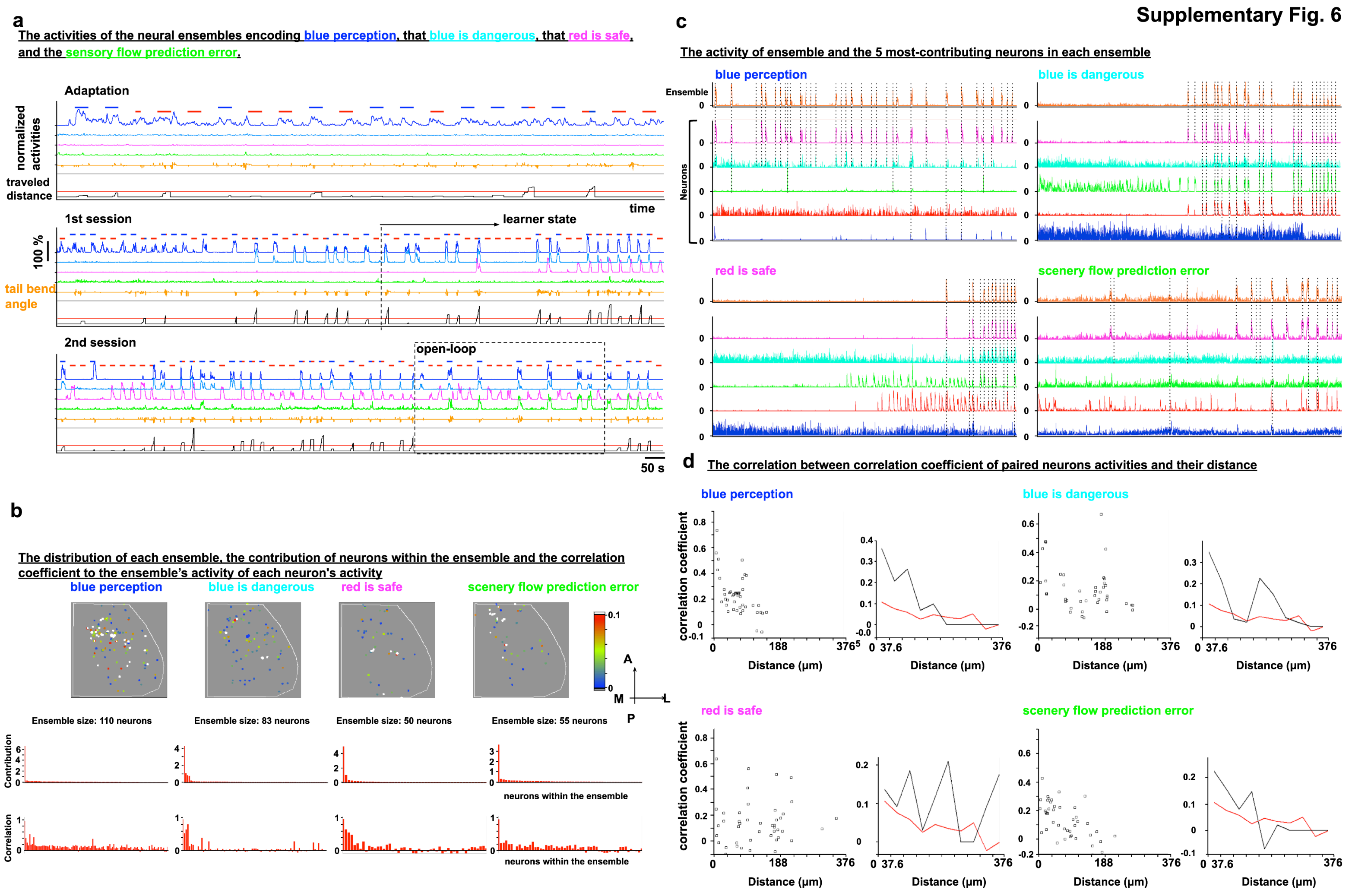
Supplementary Fig. 6
a
The activities of the neural ensembles encoding blue perception, that blue is dangerous, that red is safe, and the sensory flow prediction error.
Adaptation
normalized activities
traveled distance
time
1st session
learner state
100 %
tail bend angle
2nd session
open-loop
50 s
b
The distribution of each ensemble, the contribution of neurons within the ensemble and the correlation coefficient to the ensemble's activity of each neuron's activity
blue perception
blue is dangerous
red is safe
scenery flow prediction error
A
M
L
P
Ensemble size: 110 neurons
Ensemble size: 83 neurons
Ensemble size: 50 neurons
Ensemble size: 55 neurons
Contribution
Correlation
neurons within the ensemble
c
The activity of ensemble and the 5 most-contributing neurons in each ensemble
Ensemble
Neurons
blue perception
blue is dangerous
red is safe
scenery flow prediction error
d
The correlation between correlation coefficient of paired neurons activities and their distance
blue perception
blue is dangerous
red is safe
scenery flow prediction error
correlation coefficient
Distance (μm)

Supplementary Fig. 6. Data of Fish 2

(**a**) Activity of the identified ensembles. Notation is the same as in Fig. 3b. Blue line, activity of the ensemble encoding blue perception; cyan line, activity of the ensemble encoding that blue is dangerous; magenta line, activity of the ensemble encoding that red is safe; green line, activity of the ensemble encoding the scenery flow prediction error.

(**b**) Spatial distribution of neurons contributing to each ensemble. The color indicates the contribution of each neuron to the ensemble (top panels). A, anterior direction; P, posterior direction; L, lateral direction; M, medial direction. The contribution of neurons within each ensemble (middle panels). The correlation coefficient of activity of neurons within each ensemble and entire ensemble's activity (bottom panels).

(**c**) Activity of the ensemble (top trace) and the five most-contributing neurons in the ensemble (descending order from the top). Dotted lines indicate the timing when the neurons showed simultaneous activation with the ensemble.

(**d**) Relationship between the correlation coefficient and distance among the 10 most-contributing neurons in the ensembles. Black line denotes the averaged correlation in this fish. Red line denotes the average of 10 shuffled data (see Methods). The positively correlated neurons tended to accumulate at close distances.

Supplementary Fig. 7

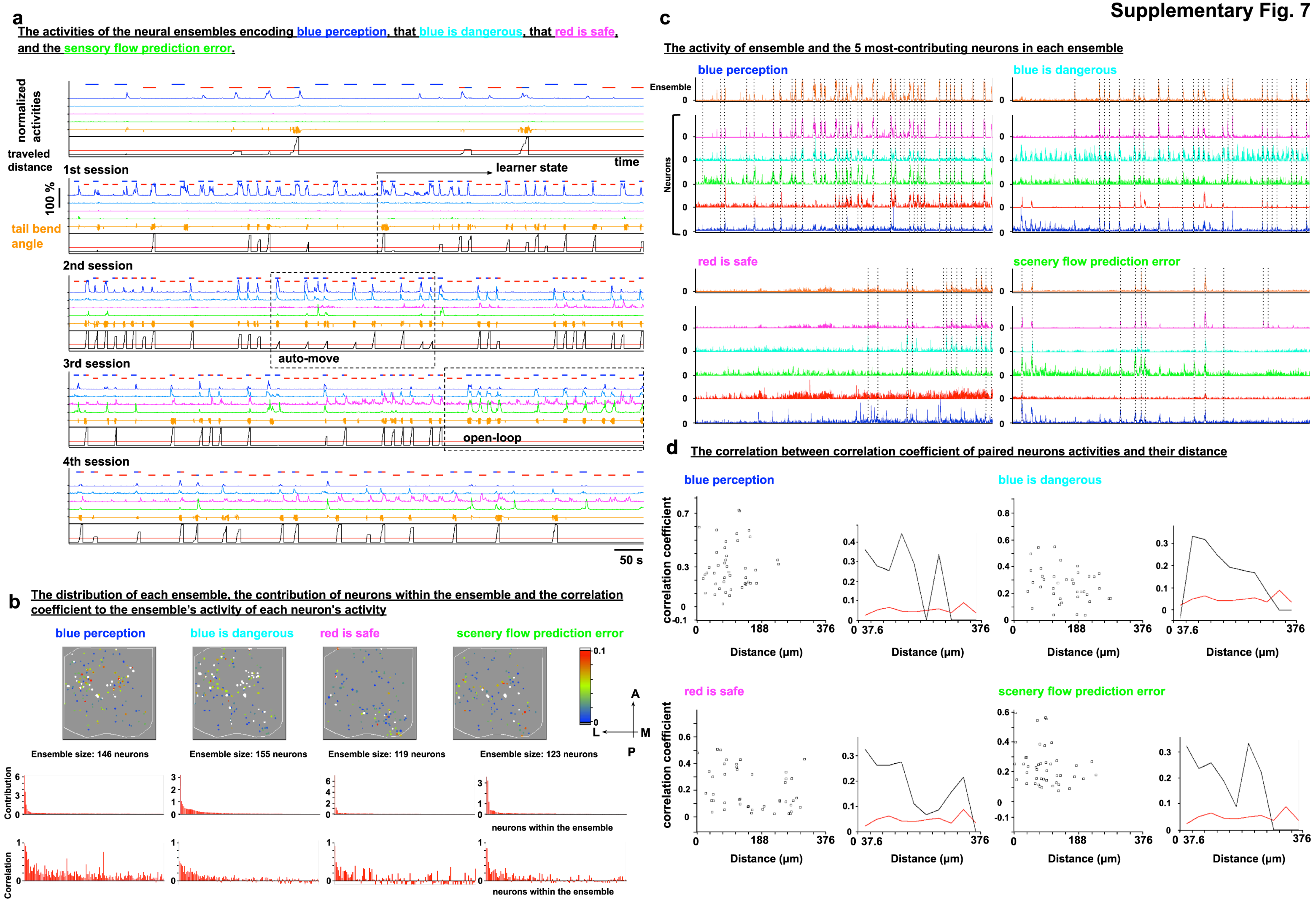

Supplementary Fig. 7. The data of Fish 3

(**a**) Activity of the identified ensembles. Notation is the same as in Fig. 3b. Blue line, activity of the ensemble encoding blue perception; cyan line, activity of the ensemble encoding that blue is dangerous; magenta line, activity of the ensemble encoding that red is safe; green line, activity of the ensemble encoding the scenery flow prediction error.

(**b**) Spatial distribution of neurons contributing to each ensemble. The color indicates the contribution of each neuron to the ensemble (top panels). A, anterior direction; P, posterior direction; L, lateral direction; M, medial direction. The contribution of neurons within each ensemble (middle panels). The correlation coefficient of activity of neurons within each ensemble and entire ensemble's activity (bottom panels).

(**c**) Activity of the ensemble (top trace) and the five most-contributing neurons in the ensemble (descending order from the top). Dotted lines indicate the timing when the neurons showed simultaneous activation with the ensemble.

(**d**) Relationship between the correlation coefficient and distance among the 10 most-contributing neurons in the ensembles. Black line denotes the averaged correlation in this fish. Red line denotes the average of 10 shuffled data (see Methods). The positively correlated neurons tended to accumulate at close distances.

Supplementary Fig.8

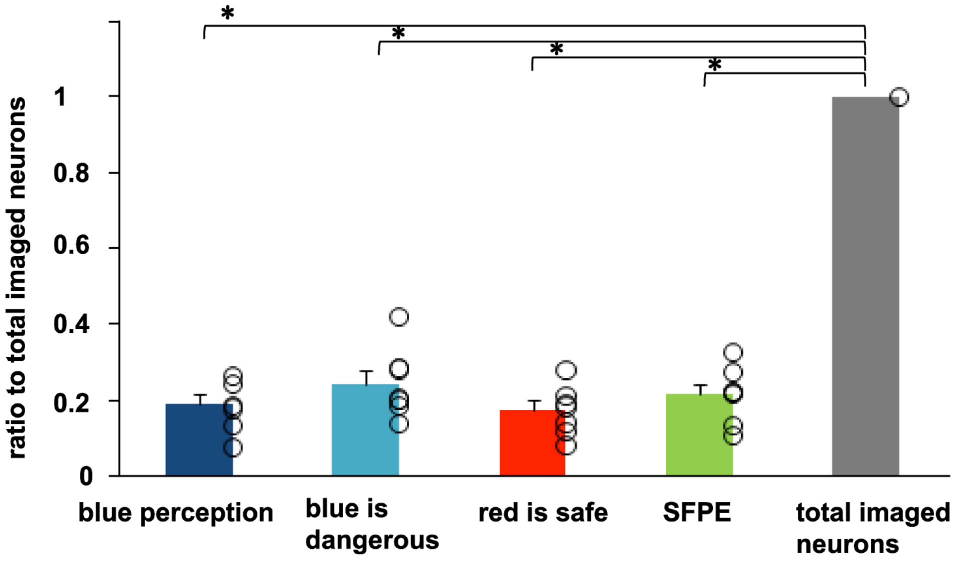

Supplementary Fig. 8 The ratio of neurons that have contribution larger than zero in each ensemble to total imaged neurons in each fish was plotted.

The seven fish which possessed all ensembles mentioned in this study were used for the calculation (Fish 1-7 in Supplementary Table 1). Bar and error bars showed averaged ratio and SEM. Each circle indicates the result of each fish. Blue perception, 0.193±0.027; blue is danger, 0.245±0.035; red is safe, 0.175±0.025; SFPE, 0.216±0.029. The number of neurons contributing to the ensemble encoding blue color perception vs the number of total imaged neurons, P=9.02x10^{-8}; the number of neurons contributing to the ensemble encoding the rule that blue is dangerous vs the number of total imaged neurons, P=6.28x10^{-7}; the number of neurons contributing to the ensemble encoding the rule that red is safe vs the number of total imaged neurons, P=4.89x10^{-8}; the number of neurons contributing to the ensemble encoding SFPE vs the number of total imaged neurons, P=1.58x10^{-7}. *P <0.05/4, two-tailed paired t-test with Bonferroni correction.

Supplementary Fig. 9

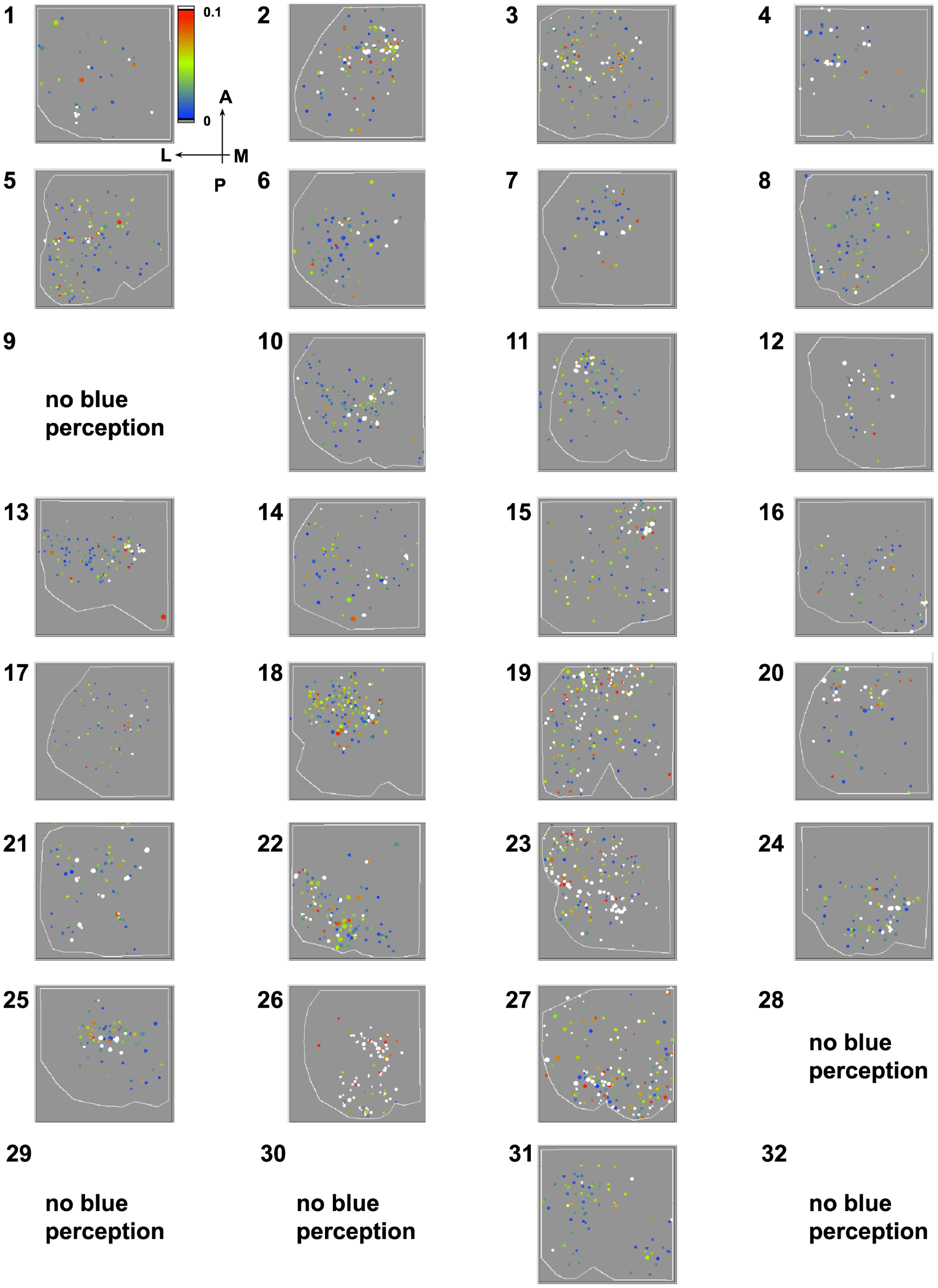

Supplementary Fig. 9. Distribution of neurons within the ensemble encoding blue perception in all fish

Each panel illustrates the distribution of neurons within the ensemble encoding blue perception. The numbers correspond to the fish number in Supplementary Table 1. The white line in each panel indicates the edge of the brain tissue. When looking at the right hemisphere, the mediolateral axis of the plot is reversed. Note that there is no single brain region where the neurons within the blue perception ensemble preferentially accumulated. A, anterior direction; P, posterior direction; L, lateral direction; M, medial direction.

Supplementary Fig. 10

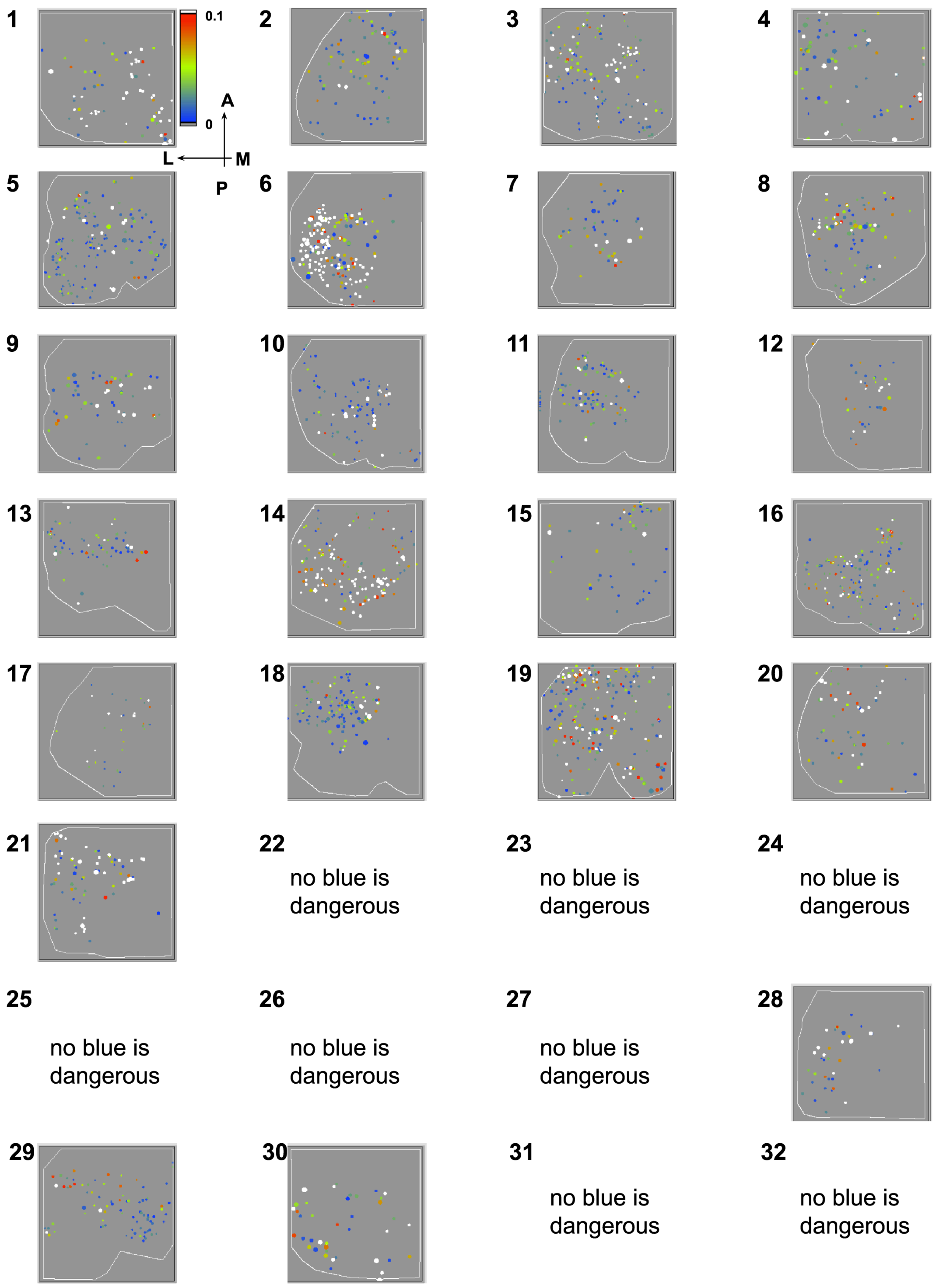

Supplementary Fig. 10. Distribution of neurons within the ensemble encoding the 'blue-dangerous' rule in all fish

Each panel illustrates the distribution of neurons within the ensemble encoding the 'blue-dangerous' rule. The numbers correspond to the fish number in Supplementary Table 1. The white line in each panel indicates the edge of the brain tissue. When looking at the right hemisphere, the mediolateral axis of the plot is reversed. Note that there is no single brain region where the neurons within this ensemble preferentially accumulated. A, anterior direction; P, posterior direction; L, lateral direction; M, medial direction.

Supplementary Fig. 11

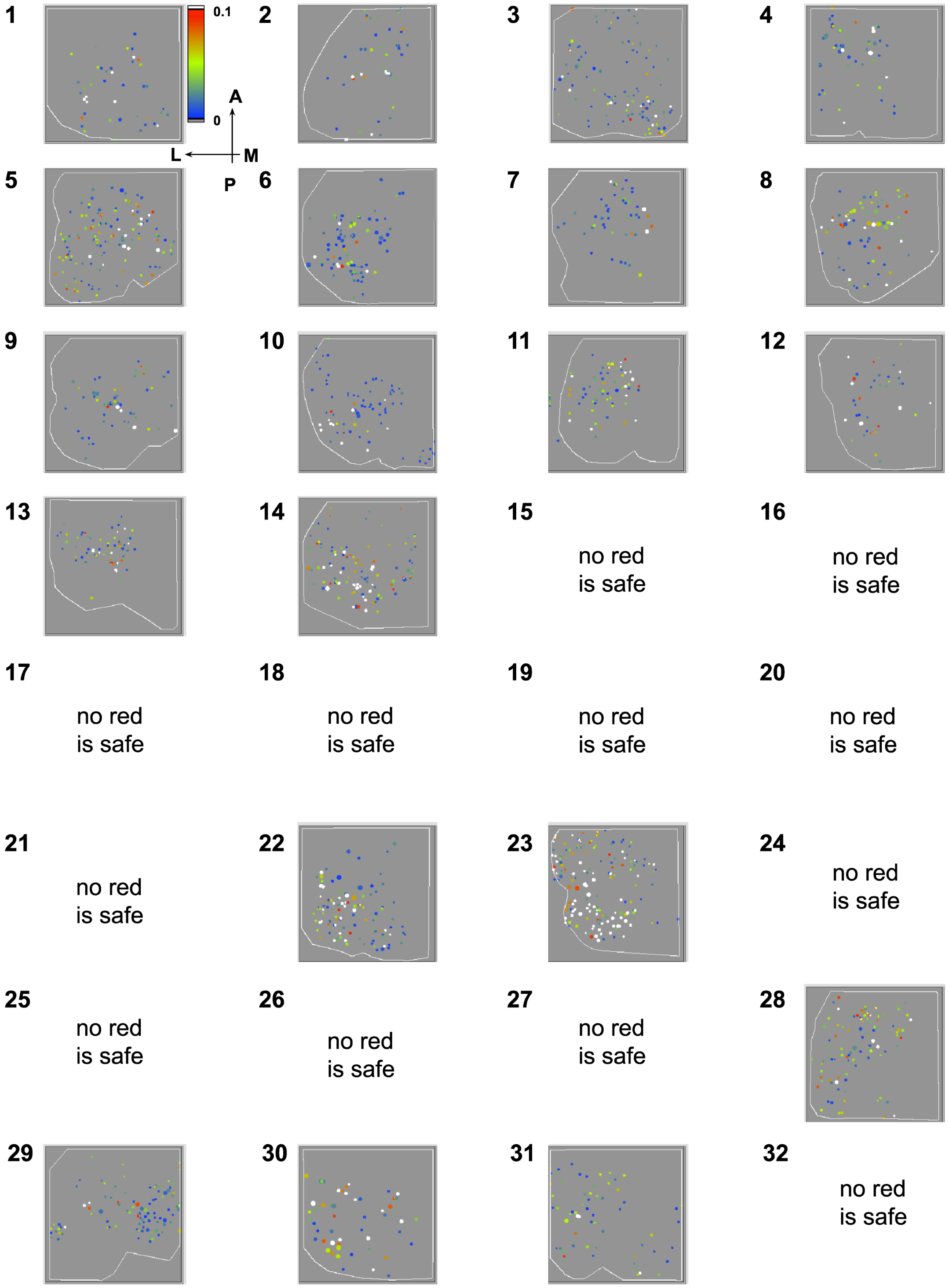

Supplementary Fig. 11. Distribution of neurons within the ensemble encoding the ‘red-safe’ rule in all fish

Each panel illustrates the distribution of neurons within the ensemble encoding the ‘red-safe’ rule. The numbers correspond to the fish number in Supplementary Table 1. The white line in each panel indicates the edge of the brain tissue. When looking at the right hemisphere, the mediolateral axis of the plot is reversed. Note that there is no single brain region where the neurons within this ensemble preferentially accumulated. A, anterior; P, posterior; L, lateral; M, medial.

Supplementary Fig. 12

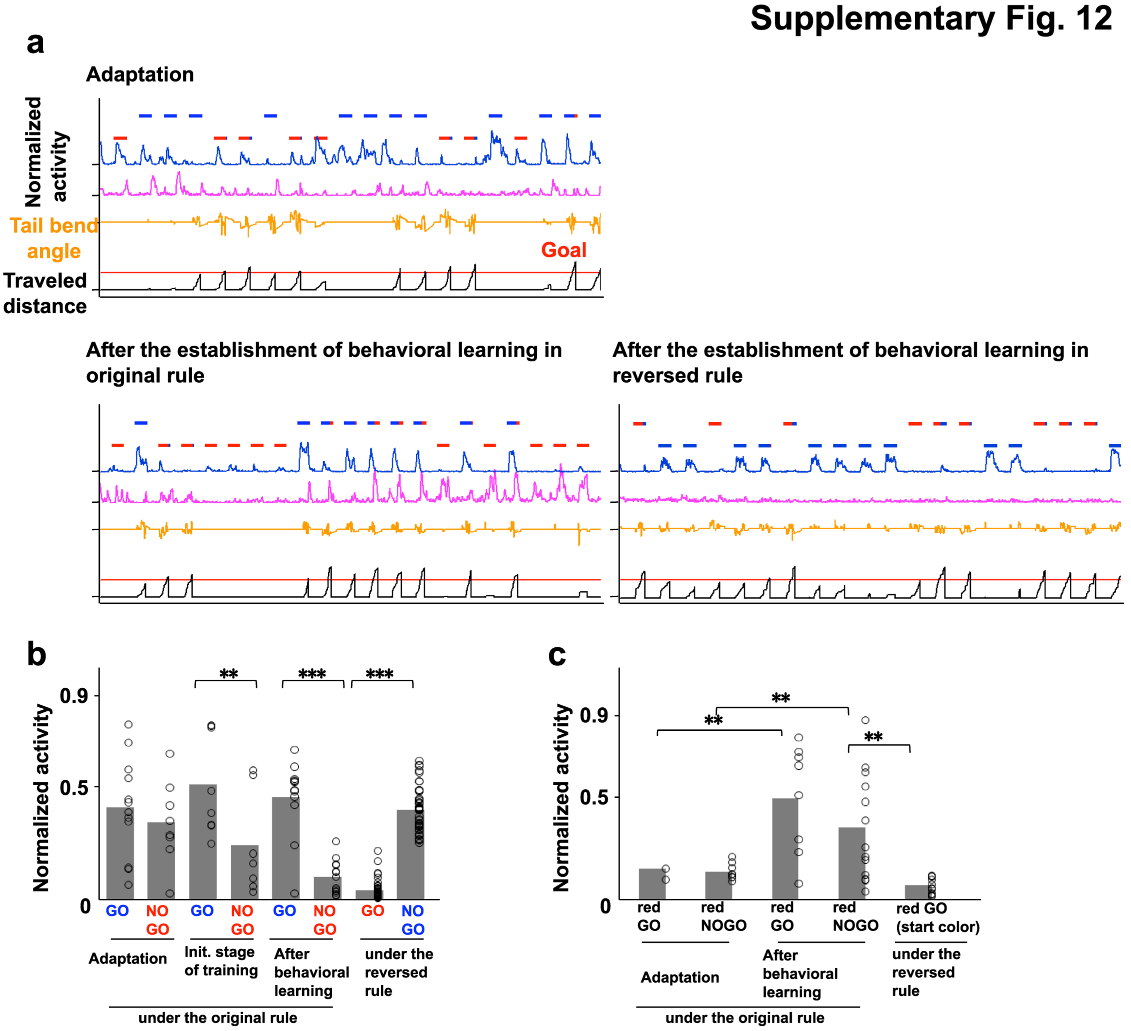

Supplementary Fig. 12. Activity of ensembles encoding blue perception and the 'red-safe' rule under the original and reversed rules.

(a) Activity of the ensembles encoding blue perception and the rule that red is safe under the original rule in the adaptation stage (upper panel), after behavioral learning was established under the original rule (bottom left panel) and the reversed rule (bottom right panel). Notation is the same as in Fig. 3b. Blue line, activity of the ensemble encoding blue perception; magenta line, activity of the ensemble encoding that red is safe under the original rule.

(b) Quantified activity of blue perception-coding ensemble in **a**. Columns and error bars: mean±SEM. Circles indicate the peak value in each GO or NOGO trial.

(c) Quantified activity of the ensemble encoding the rule that red is safe in **a**. Columns and error bars: mean±SEM. Circles indicate the peak value when fish was in red region in each GO or NOGO trial.

The ensemble that showed increased activity when fish perceived blue color under the original rule showed increased activity in NOGO trials under the reversed rule (blue line). Fish started to perceive blue color, confirming that this ensemble encodes the perception of blue color. The activity of ensemble that showed increased activity when fish perceived red color in repeated trials under the original rule did not show increased activity when fish perceived red color in repeated trials under the reversed rule (magenta line), suggesting that this ensemble encoded the 'red-safe' rule.

Supplementary Fig. 13

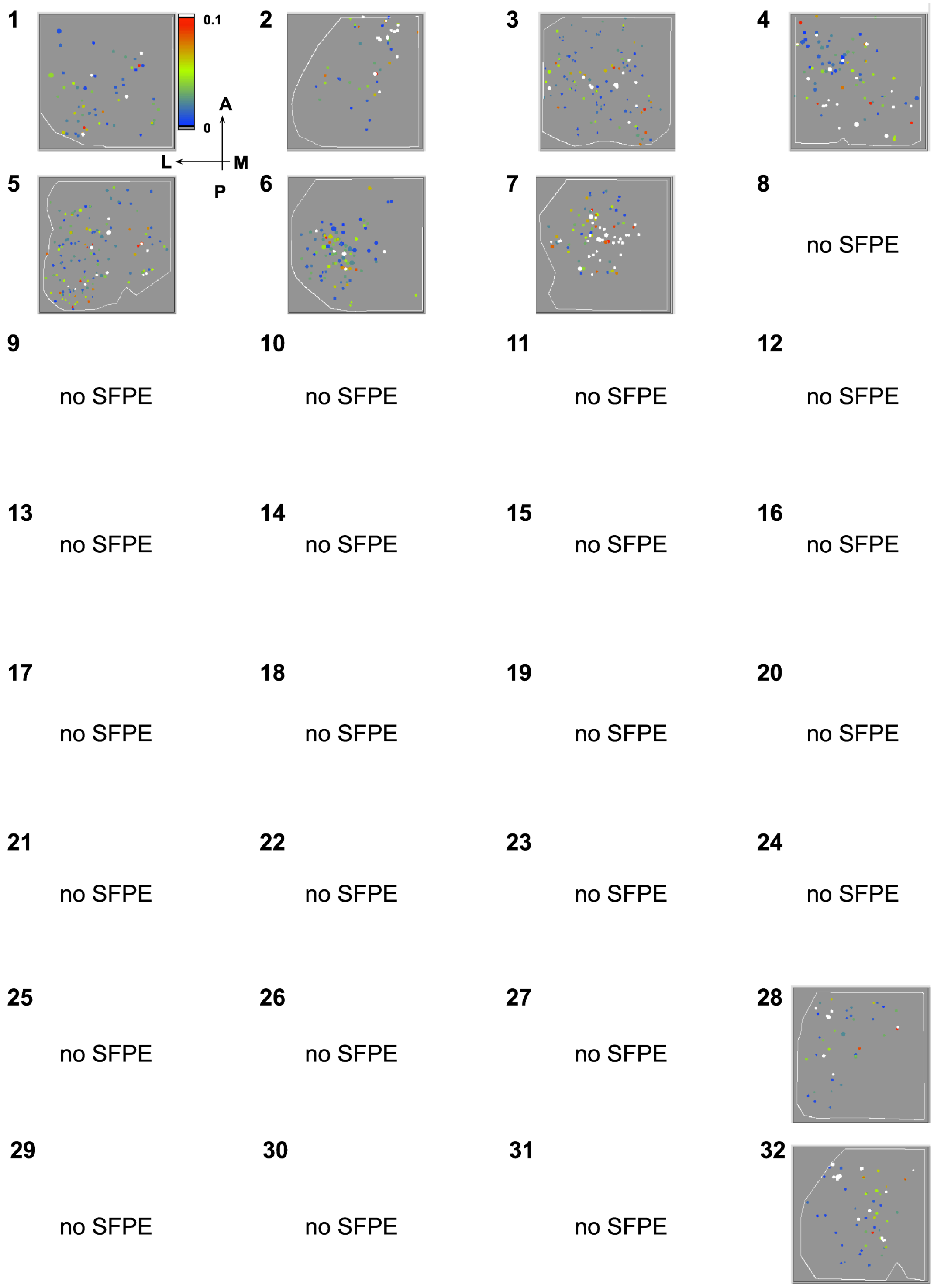

Supplementary Fig. 13. Distribution of neurons within the ensemble encoding the scenery flow prediction error in all fish

Each panel illustrates the distribution of neurons within the ensemble encoding the scenery flow prediction error. Numbers correspond to the fish number in Supplementary Table 1. The white line in each panel indicates the edge of the brain tissue. When looking at the right hemisphere, the mediolateral axis of the plot is reversed. Note that there is no single brain region where the neurons within this ensemble preferentially accumulated. A, anterior direction; P, posterior direction; L, lateral direction; M, medial direction.

Supplementary Fig. 14

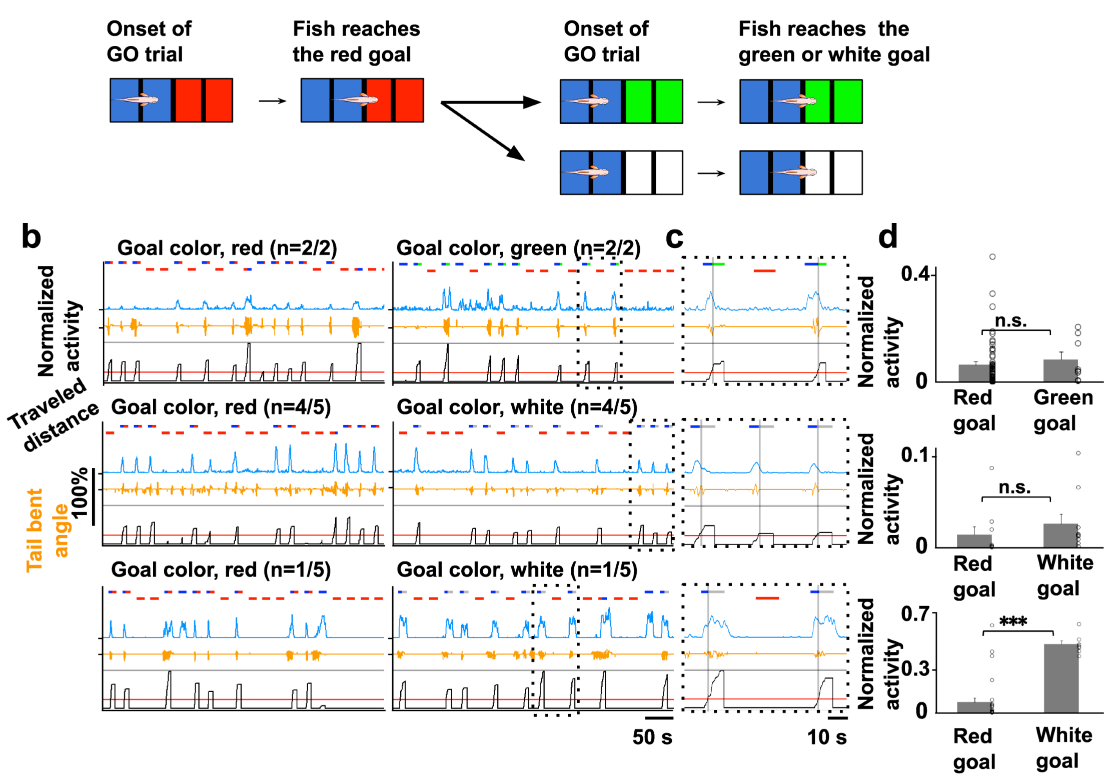

Supplementary Fig. 14. The putatively color value-encoding ensemble is unlikely to encode the prediction error with respect to the future favorable goal color

Notations in the figures are all the same as in Fig. 3b.

(**a**) Schema of the goal color change experiment from the onset of the GO trial. In the GO trials, we changed the goal color from red to green or white from the onset of the GO trials after behavioral learning was established.

(**b**) Activity of ensembles that exhibited increased activity when fish perceived blue color as training with the original rule proceeded. Upper panels, the activity when goal color was changed to green from red; middle and bottom panels, the activity when goal color was changed to white.

(**c**) Enlarged views of the boxed areas in (b). Vertical gray line indicates the timepoint when fish reached the goal.

(**d**) Comparison of the activity after reaching the goal when the goal color was red and green or white. Columns and error bars: mean±SEM. Upper panel: red-goal, 0.064±0.012; green goal, 0.084±0.029; middle panel: red-goal, 0.093±0.034; white goal, 0.023±0.008; bottom panel: red-goal, 0.072±0.03; white goal, 0.48±0.023. Circles indicate the peak value after reaching the goal in each GO trial. ***P=3.75x10^{-8}. n.s.,

not significant. Two-tailed unpaired *t*-test.

In three of the 32 fish that were analyzed in Fig. 2-7, we changed the goal color to green before or after the open-loop experiments and, in two of the three fish, we observed the ensemble that was activated when fish perceived blue color as training proceeded. In these two fish, the ensemble activity decreased when the fish reached the green goal (Supplementary Fig. 14b-d, upper panels). We also changed the goal color to white in eight of the 32 fish and, in five of these eight fish, we observed the ensembles that were activated when fish perceived blue colors as training proceeded. The activity of this ensemble returned to baseline after reaching the goal in four of the five fish (Supplementary Fig. 14b-d, middle panels). In the remaining one fish, the ensemble's activity did not decrease, even if the fish reached the goal and perceived the white goal color (Supplementary Fig. 14b-d, bottom panels).

In six of the seven fish that experienced goal color change, the ensemble's activity decreased when the fish escaped from the blue region regardless of the changed goal color (green or white). In only one of the seven fish did the ensemble activity remain, even when the fish escaped from the blue region and perceived the changed goal color (white). Taken together, the ensemble, which showed increased activity when fish perceived blue color as training proceeded, was in most cases unlikely to encode the prediction error.

Supplementary Fig. 15

a

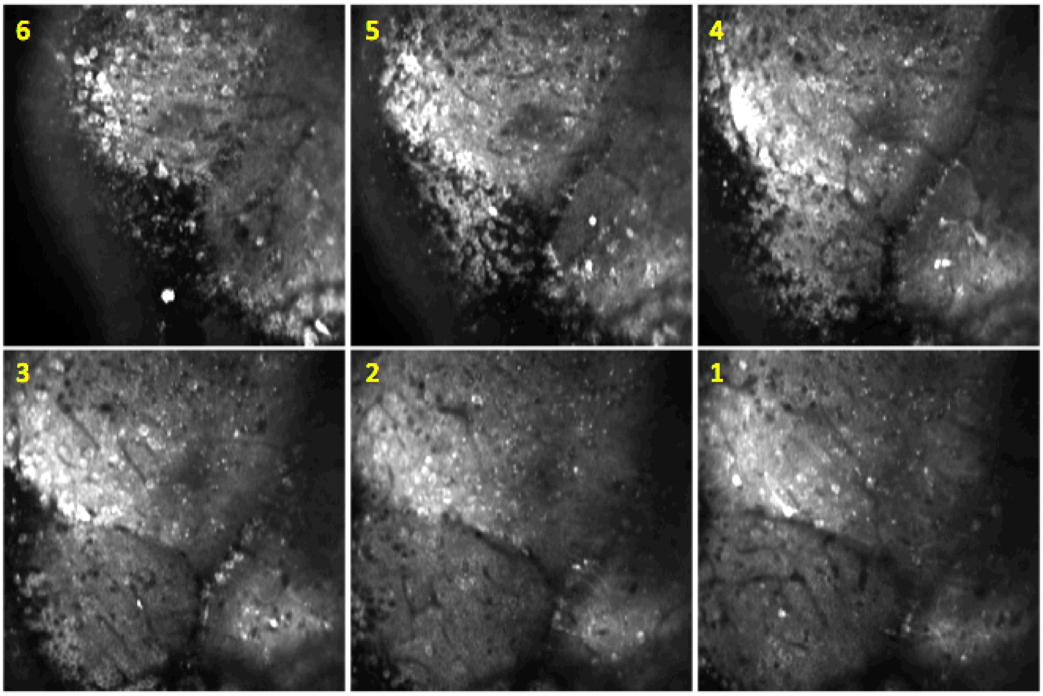

b

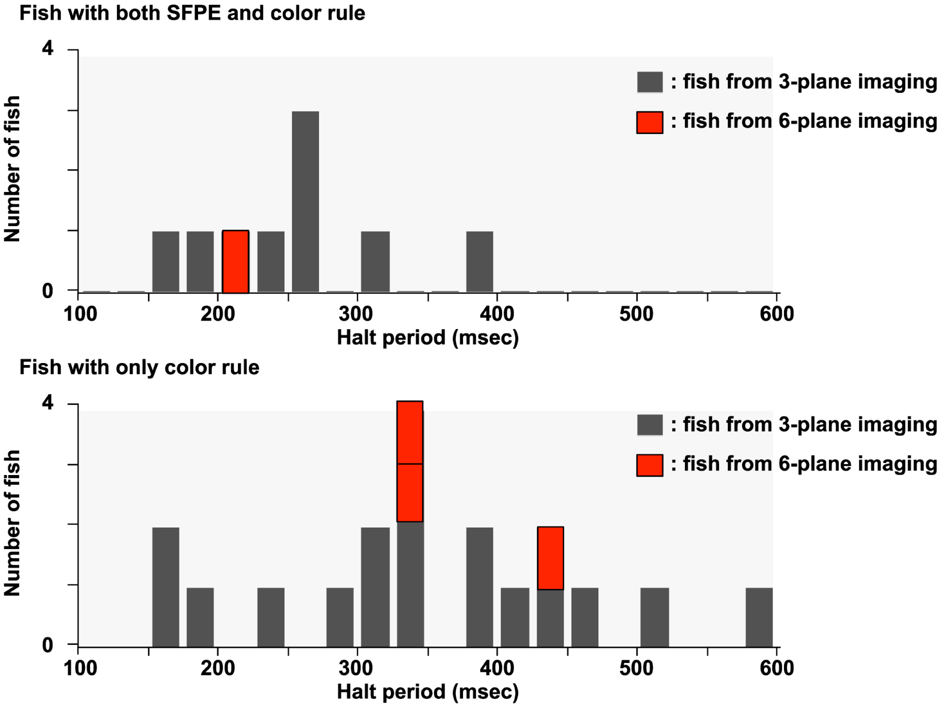

Supplementary Fig. 15. Deep imaging and distribution of halt periods

(**a**) Imaged slices with 6-plane imaging. 6: shallowest slice, 1: the deepest slice.

The image is left hemisphere containing the lateral zone, the central zone, and the medial zone of dorsal telencephalon.

(**b**) Halt periods in two groups. Upper panel refers to fish with both scenery flow prediction error and color rule (upper panel). Lower panels refer to fish with only color rule. Red rectangles indicate the data from deep imaging data. Note that newly obtained fish with deeper imaging which had scenery flow prediction error showed shorter halt periods than those without scenery flow prediction error (red rectangles).

Supplementary Fig. 16

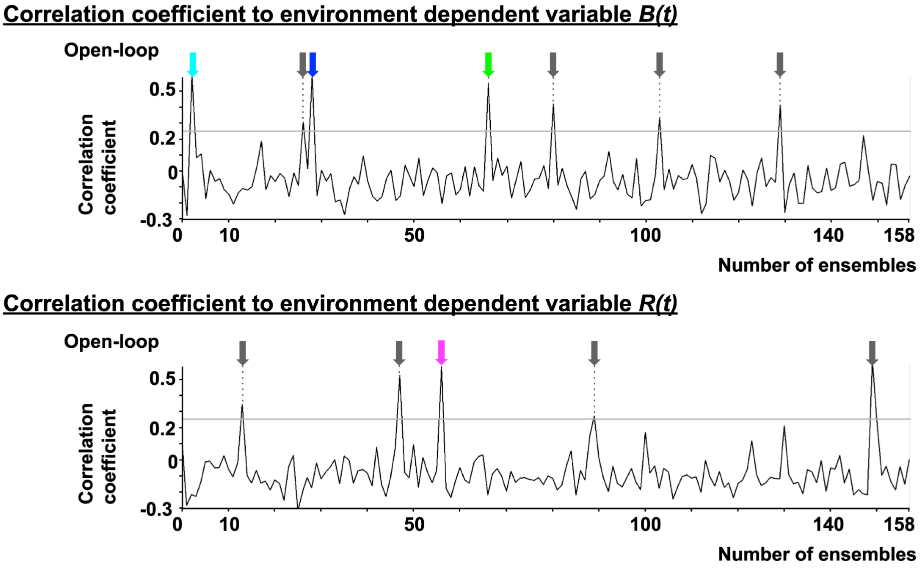

Supplementary Fig. 16. The semi-automatized procedure for the selection of the ensembles encoding the information we focused on in Fish 1 in Supplementary Table 1

This figure shows the result of application of the semi-automatic selection procedure to the original neural activity data of Fish1 in Supplementary Table1 as an example. The upper and bottom panels show the result of correlation coefficient between NMF ensembles and environment-dependent variables, *B(t)* and *R(t)* under the open loop condition, respectively. If the correlation coefficient to *B(t)* was higher than 0.25 (a threshold for the selection shown in gray line), the ensembles were regarded as candidates of the ensembles encoding blue perception, the rule that blue is dangerous or putative SFPE. Among the seven candidates which showed correlation coefficient higher than 0.25, as shown in blue, cyan and green arrows, three ensembles met the criteria in the flow chart in Supplementary Fig. 19 (upper panel). These blue, cyan and green ensembles were regarded as blue perception, the rule that that blue is dangerous and putatively SFPE-encoding ensembles, respectively. Other four ensembles, as shown in gray arrows, were abandoned, as they did not show the specificity in activation to blue color under the closed loop condition or did not show the constant activation in GO trials under the open-loop condition. For identification of the ensemble encoding the rule that red is safe, we also performed the selection based on the flow chart in Supplementary Fig. 20. In the Fish1, five NMF ensembles showed higher correlation coefficient than 0.25 (the threshold for the selection shown in gray line). Among these

five ensembles, as shown in magenta arrow, one ensemble met the criteria in the flow chart in Supplementary Fig. 20. The other four ensembles were abandoned, as they did not show specificity in the activation to red color under the closed loop condition or only showed increased activity to red either at the end of GO trials or during NOGO trials.

Supplementary Fig. 17

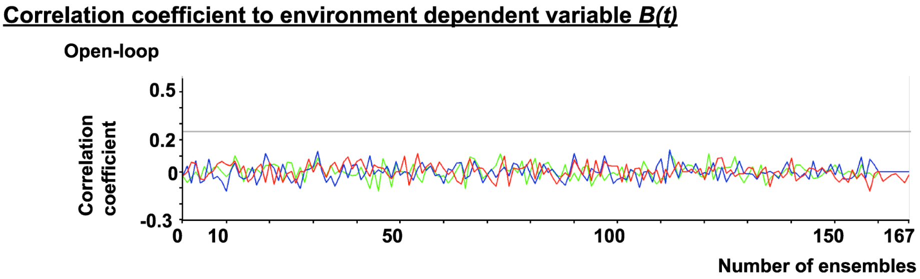

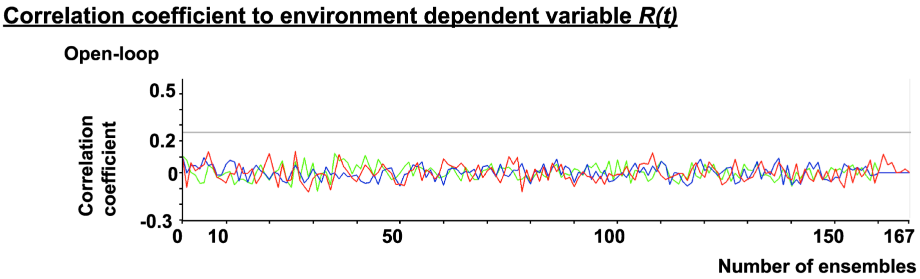

Supplementary Fig. 17. No NMF ensembles calculated from the randomly shuffled neural activity from Fish1 data showed were selected as candidates as the ensembles of our interest by the semi-automatized screening.

We examined whether the ensembles we focused on (*e.g.* the ensembles encoding blue perception, color rules and putative SFPE) could be obtained by chance. For this, we performed the same analysis based on Supplementary Fig. 16 using the NMF ensembles generated by random shuffling of the original data in each fish (see Methods). This figure shows the correlation coefficient of NMF ensembles calculated from the shuffled neural activity from the original data of Fish1 in Supplementary Table 1 to *B(t)*: upper panel and *R(t)*: bottom panel in the open loop condition. Three sets of shuffled neural activity were prepared from the neural activity in Fish1. The abscissa and ordinate indicate the ordering number of the ensembles and the correlation coefficient, respectively. Red, green and blue color lines indicate the results from three independently shuffled neural activity sets. As a result, no ensemble showed higher value than the threshold value (0.25, gray line in each panel), and no ensemble was selected. These results show that no ensembles of our interest were obtained by chance.

Supplementary Fig. 18

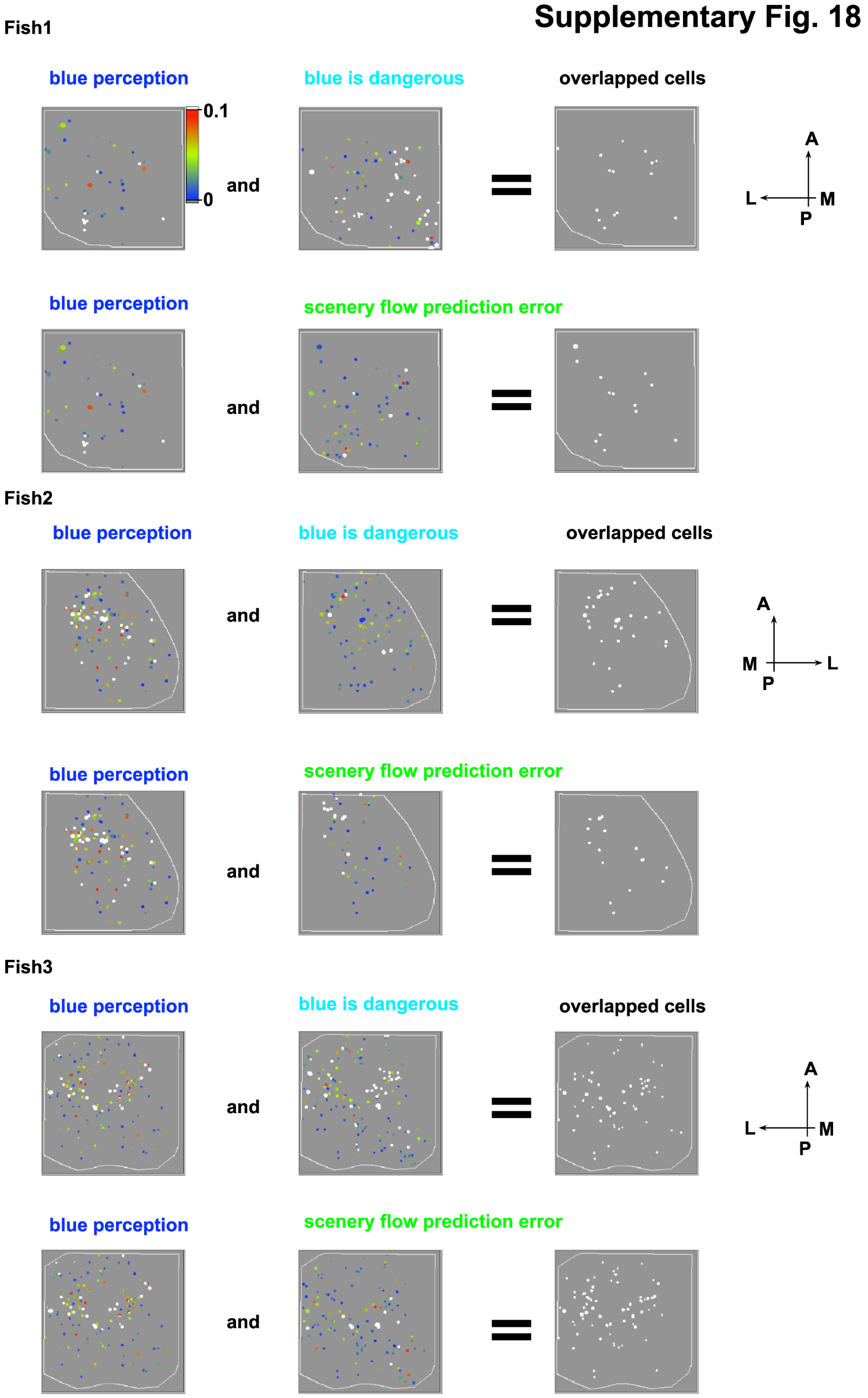

Supplementary Fig. 18. Overlapping neurons between ensembles encoding blue perception and the 'blue-dangerous' rule and the scenery flow prediction error in Fish 1-3. A, anterior direction; P, posterior direction; L, lateral direction; M, medial direction.

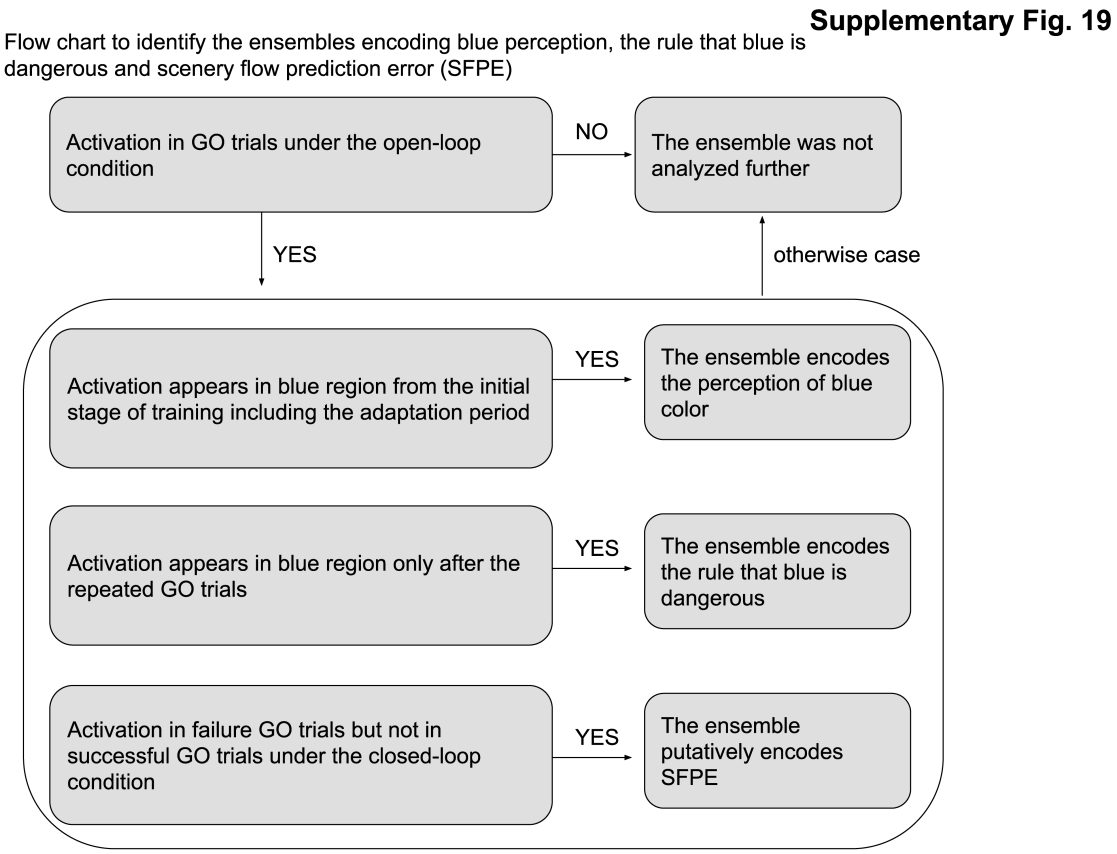

Supplementary Fig. 19. The flow chart for identification the ensembles encoding blue perception, the rule that blue is dangerous and putative SFPE.

Supplementary Fig. 20

Flow chart to identify the ensembles encoding the rule that red is safe

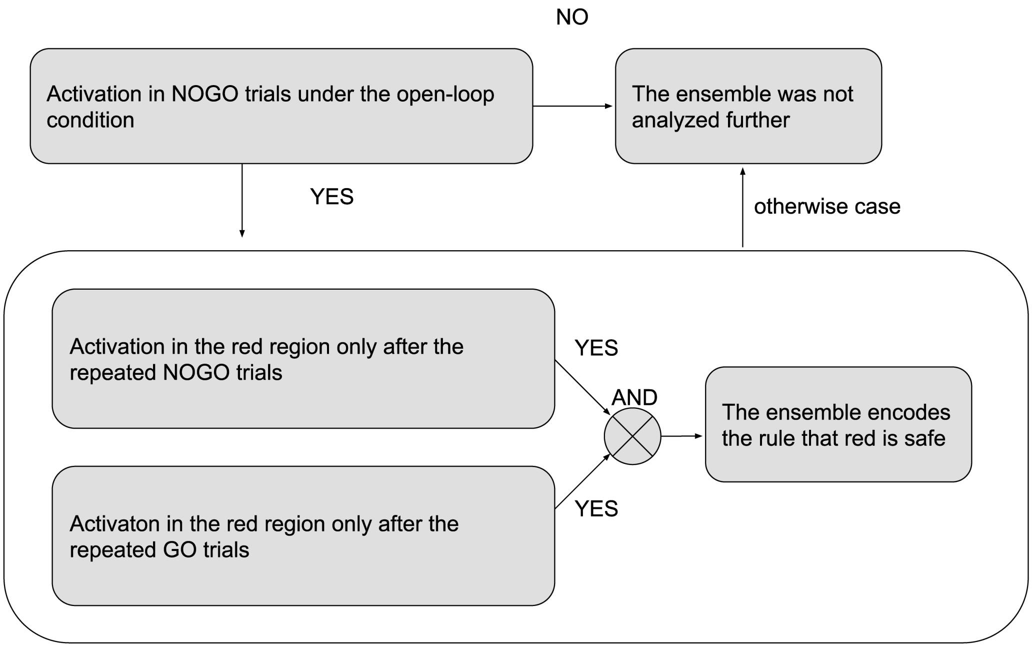

Supplementary Fig. 20. The flow chart for identification the ensembles encoding the rule that red is safe.

Supplementary Fig. 21

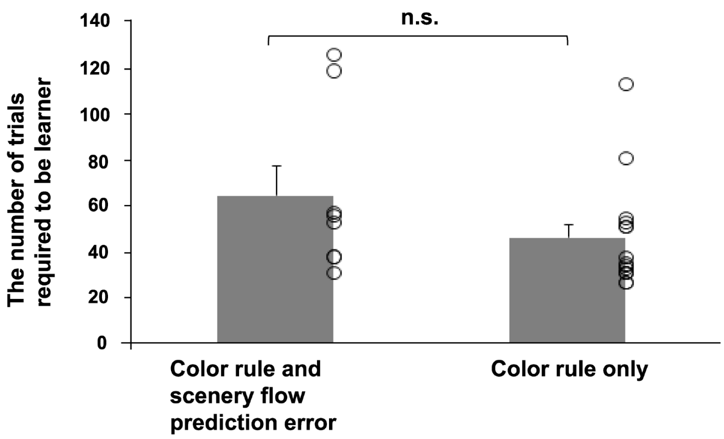

Supplementary Fig. 21 Fish with, and fish without the SFPE ensemble required a comparable number of trials until behavioral learning was established.

Comparison of the number of trials until behavioral learning was established between two groups. The number of fish which has color rule-coding and putative SFPE ensembles is 8 and that of fish which has only color rule-coding ensemble is 16. Columns and error bars: mean±SEM. Each circle indicates the value in each fish. Left: *P*=0.147884. two-tailed unpaired *t*-test. n.s., not significant.

Supplementary Table 1. Summary of generated ensembles in all learner fish

	blue perception	blue is dangerous	red is safe	scenery flow prediction error
fish1	(+)	(+)	(+)	(+)
fish2	(+)	(+)	(+)	(+)
fish3	(+)	(+)	(+)	(+)
fish4	(+)	(+)	(+)	(+)
fish5	(+)	(+)	(+)	(+)
fish6	(+)	(+)	(+)	(+)
fish7	(+)	(+)	(+)	(+)
fish8	(+)	(+)	(+)	(-)
fish9	(-)	(+)	(+)	(-)
fish10	(+)	(+)	(+)	(-)
fish11	(+)	(+)	(+)	(-)
fish12	(+)	(+)	(+)	(-)
fish13	(+)	(+)	(+)	(-)
fish14	(+)	(+)	(+)	(-)
fish15	(+)	(+)	(-)	(-)
fish16	(+)	(+)	(-)	(-)
fish17	(+)	(+)	(-)	(-)
fish18	(+)	(+)	(-)	(-)
fish19	(+)	(+)	(-)	(-)
fish20	(+)	(+)	(-)	(-)
fish21	(+)	(+)	(-)	(-)
fish22	(+)	(-)	(+)	(-)
fish23	(+)	(-)	(+)	(-)
fish24	(+)	(-)	(-)	(-)
fish25	(+)	(-)	(-)	(-)
fish26	(+)	(-)	(-)	(-)
fish27	(+)	(-)	(-)	(-)
fish28	(-)	(+)	(+)	(+)
fish29	(-)	(+)	(+)	(-)
fish30	(-)	(+)	(+)	(-)
fish31	(+)	(-)	(+)	(-)
fish32	(-)	(-)	(-)	(+)
	(+) = 27 out of 32	(+) = 24 out of 32 fish	(+) = 20 out of 32 fish	(+)= 9 out of 32 fish

reversal learning	blue perception	blue is dangerous	red is safe	red is dangerous	blue is safe
fish33	(+)	(+)	(+)	(+)	(-)
fish34	(+)	(+)	(+)	(+)	(+)
fish35	(+)	(+)	(-)	(+)	(+)
fish36	(+)	(-)	(-)	(-)	(-)
fish37	(-)	(+)	(-)	(-)	(-)
	(+) = 4 out of 5 fish	(+) = 4 out ouf 5 fish	(+) = 2 out of 5 fish	(+) = 3 out of 5 fish	(+) = 2 out of 5 fish

Supplementary Table 2. Summary of results of identified ensembles in surface and deep planes

	blue perception	blue is dangerous	red is safe	scenery flow prediction error
FishA<br>surface<br>deep	 (−)<br>(−)	 (+)<br>(+)	 (+)<br>(+)	 (+)<br>(−)
FishB<br>surface<br>deep	 (+)<br>(−)	 (−)<br>(+)	 (−)<br>(−)	 (−)<br>(−)
FishC<br>surface<br>deep	 (+)<br>(+)	 (+)<br>(+)	 (+)<br>(+)	 (−)<br>(−)
FishD<br>surface<br>deep	 (+)<br>(+)	 (+)<br>(+)	 (+)<br>(−)	 (−)<br>(−)
