# Supplementary material for: Zebrafish capable of generating future state prediction error show improved active avoidance behavior in virtual reality
Source: Nat Commun. 2021 Sep 29;12:5712. doi: 10.1038/s41467-021-26010-7 (PMC8481257; doi:10.1038/s41467-021-26010-7)
Supplement: Supplementary file 3 — Description of Additional Supplementary Files. [file 41467_2021_26010_MOESM3_ESM.pdf]

Description of Additional Supplementary Files

File Name: Supplementary Movie 1
Description: Telencephalic neuronal activity during the task in virtual reality
Movie of the Calcium imaging of neural activity in the same frames as provided in Fig. 1d. The movie shows imaged neural activity from the left hemisphere in three focal planes. From the left to the right, images of the surface, middle and deep planes were presented. Anterior to top; lateral to left; medial to right.

File Name: Supplementary Movie 2
Description: Using pigment-deficient fish enabled us to image the telencephalic neuronal activity without opening the skull. Movie of the tethered zebrafish focusing on the telencephalic region. The fish exhibited saccade eye and gill movement.

File Name: Supplementary Movie 3
Description: A representative successful GO trial to show actual tail beats and feedbacked scenery movement. After 15 sec of the interval period, the GO trial started and the colors of the surrounding walls changed from white to blue. Then, fish beat the tail and escaped into the red goal region.

File Name: Supplementary Data 1
Description: Traveled distance of all 24 fish. Each slide shows the travelled distance of individual fish in all GO trials. The first eight slides show the traveled distance of eight fish with both ensembles encoding the 'blue- dangerous' rule and encoding the scenery flow prediction error. The other remaining slides show the traveled distance of fish with only the ensemble encoding 'blue-dangerous'. Fish number in each slide corresponds to that in Supplementary Table 1. The fish were aligned according to the duration of halts, both among the first eight fish and among the other remaining fish. Each boxed area indicates the period when additional manipulation was added. Some manipulations, such as the one indicated "Auto Move", are not mentioned in this work. Red line indicates the distance at the border where the presented color changed.
