# Supplementary material for: Zebrafish capable of generating future state prediction error show improved active avoidance behavior in virtual reality
Source: Nat Commun. 2021 Sep 29;12:5712. doi: 10.1038/s41467-021-26010-7 (PMC8481257; doi:10.1038/s41467-021-26010-7)
Supplement: Supplementary file 7 — Supplementary Data 1. [file 41467_2021_26010_MOESM7_ESM.zip › Supplementary Data 1.pptx]

## Slide 1
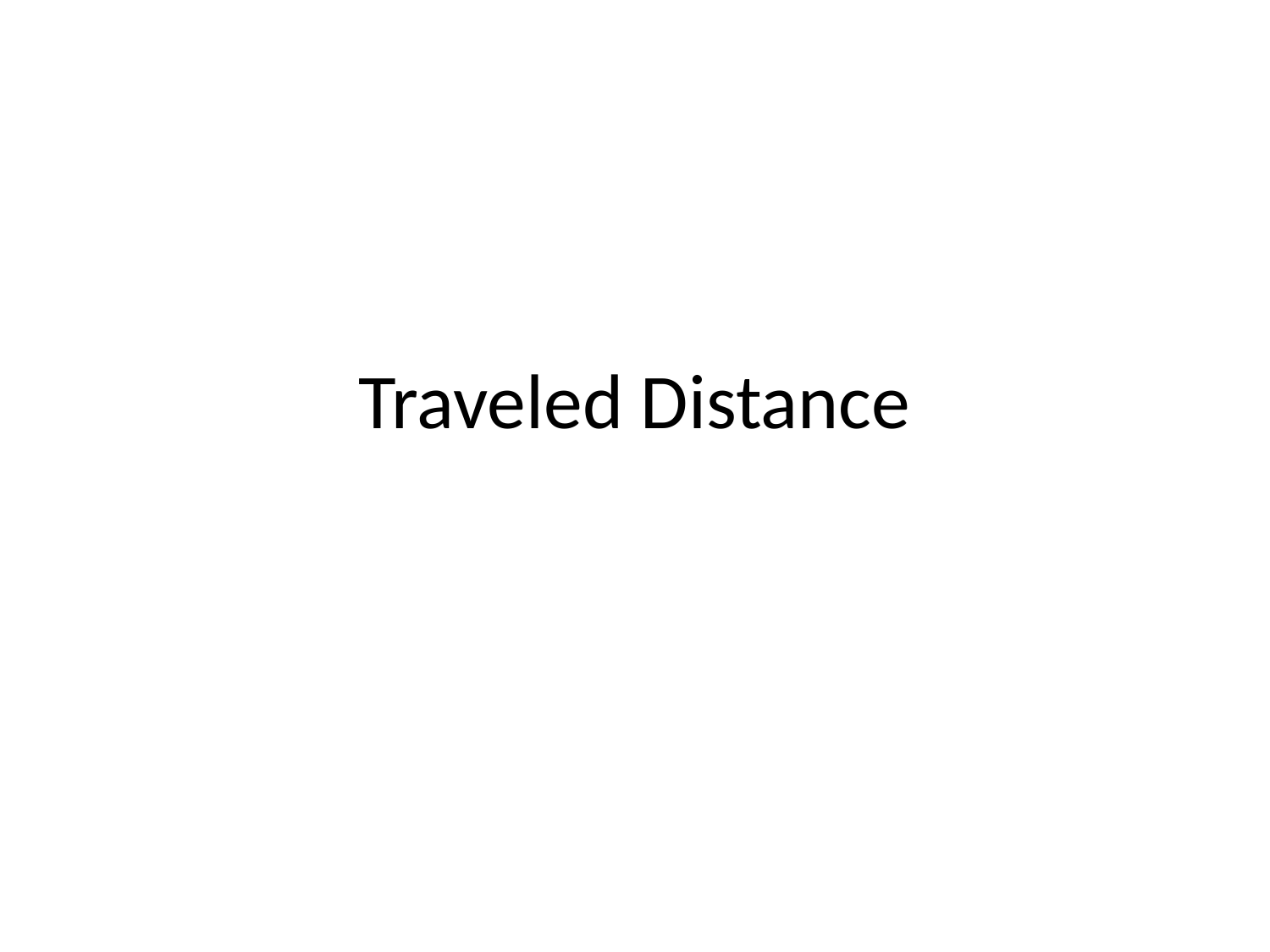

# Traveled Distance

## Slide 2
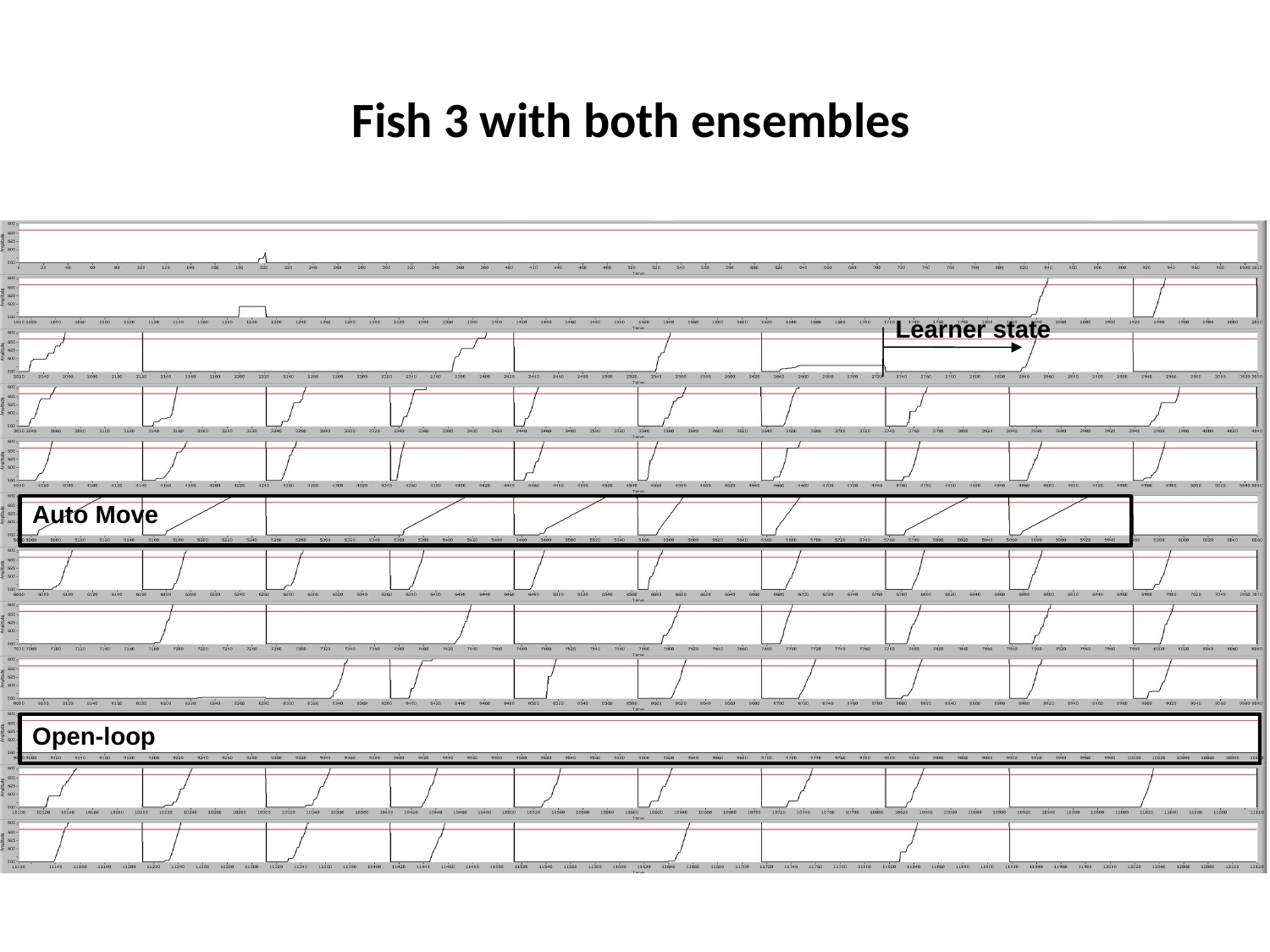

# Fish 3 with both ensembles
Learner state
Auto Move
Open-loop

## Slide 3
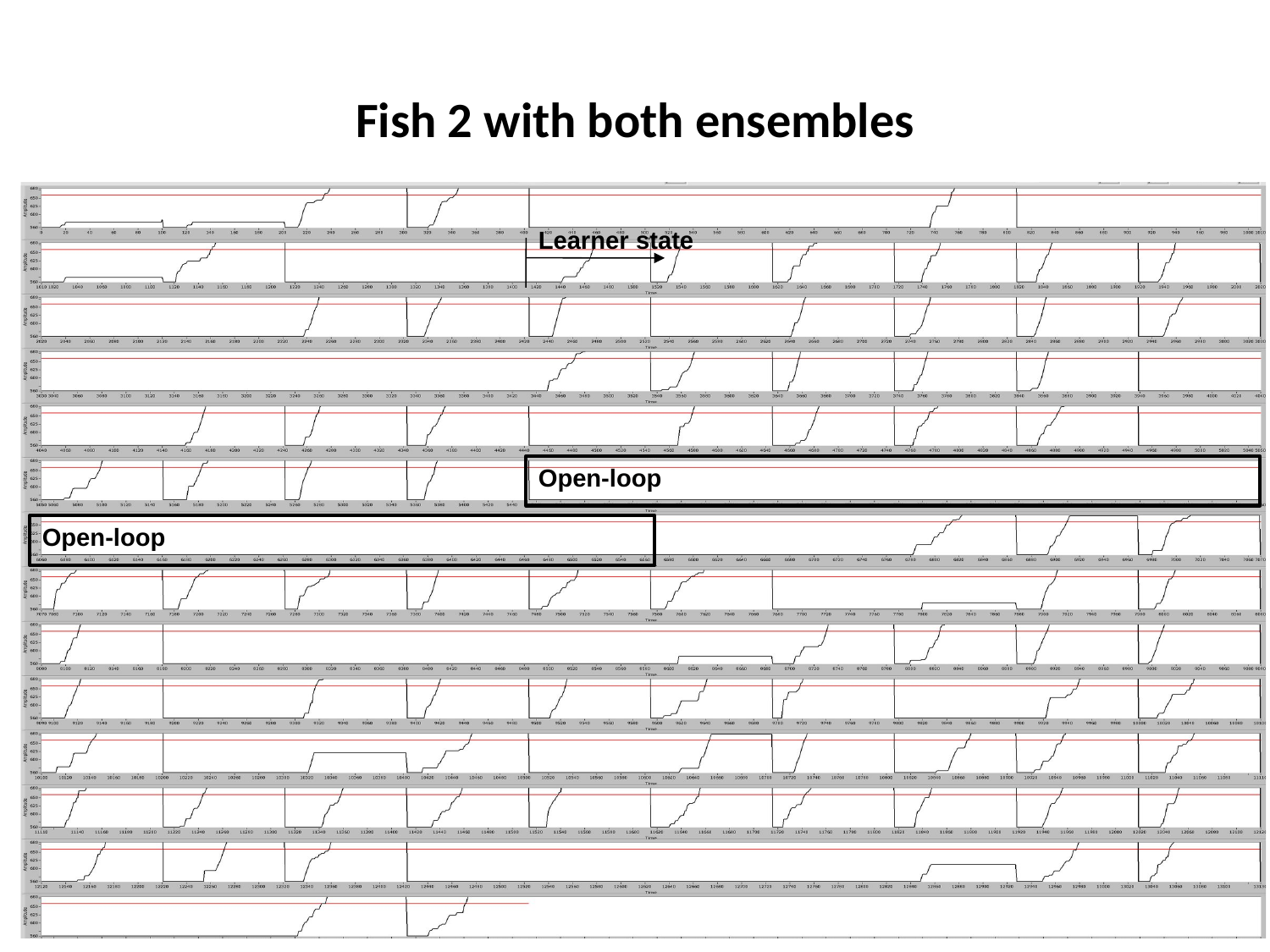

# Fish 2 with both ensembles
Learner state
Open-loop
Open-loop

## Slide 4
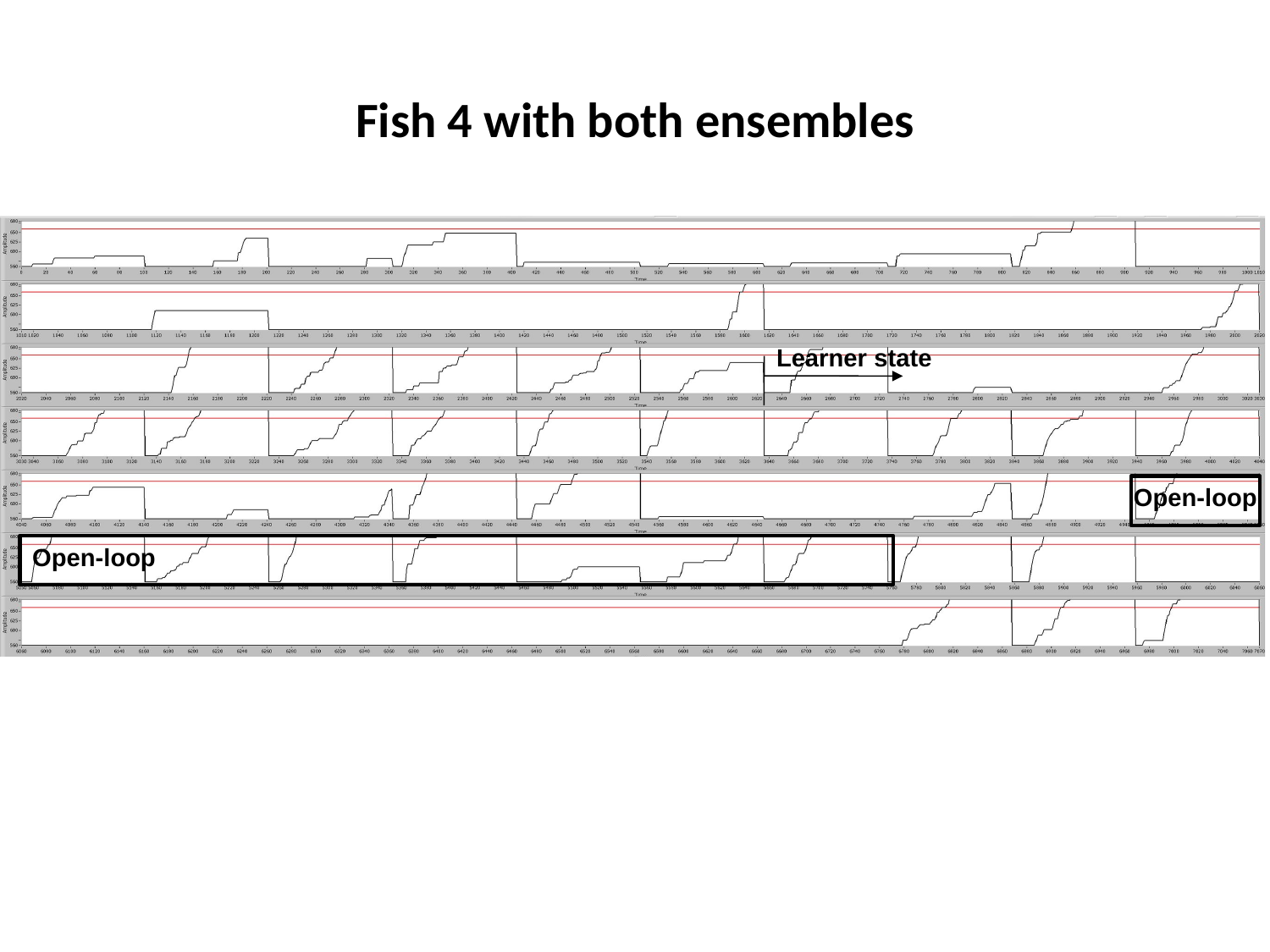

# Fish 4 with both ensembles
Learner state
Open-loop
Open-loop

## Slide 5
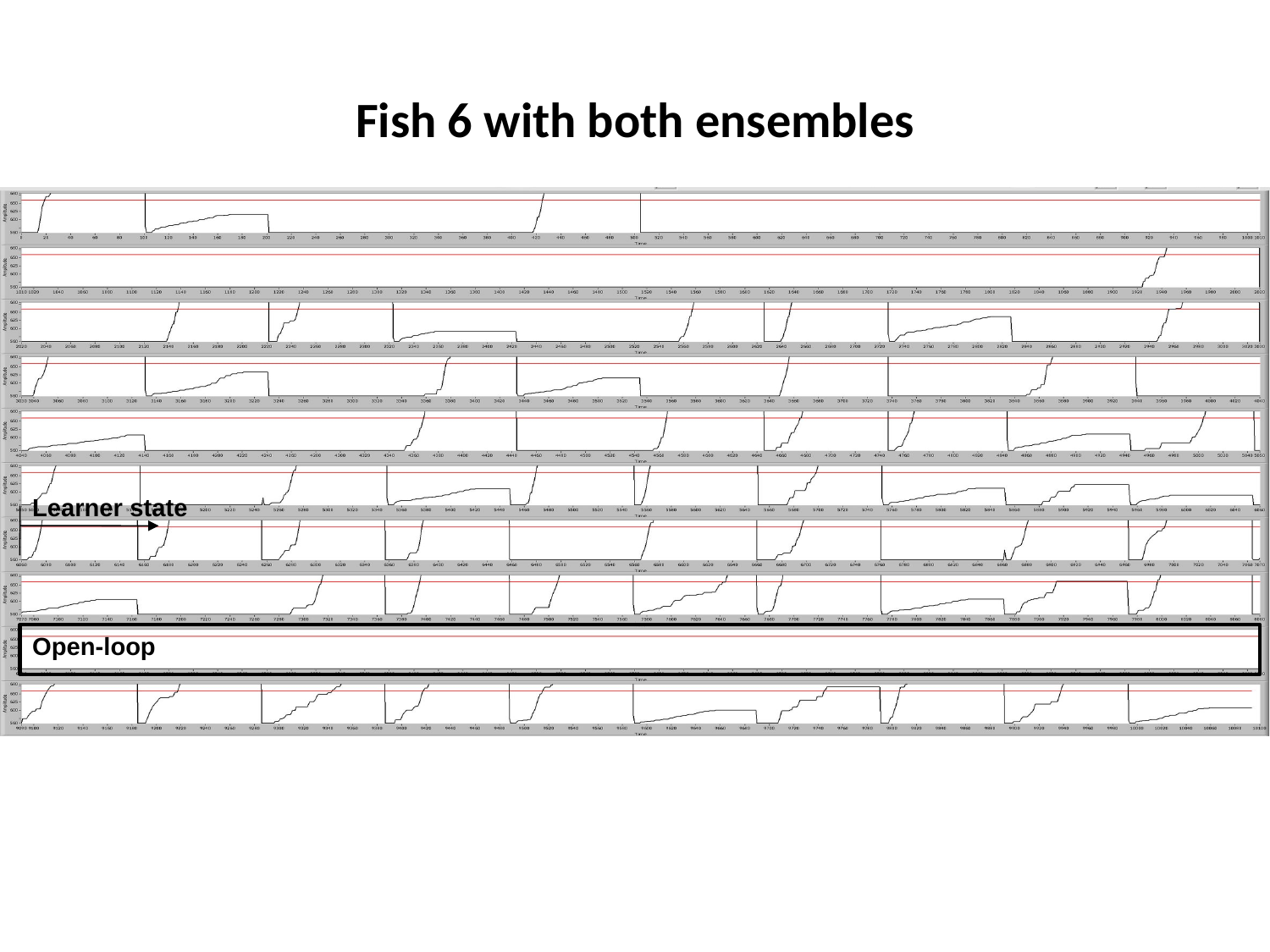

# Fish 6 with both ensembles
Learner state
Open-loop

## Slide 6
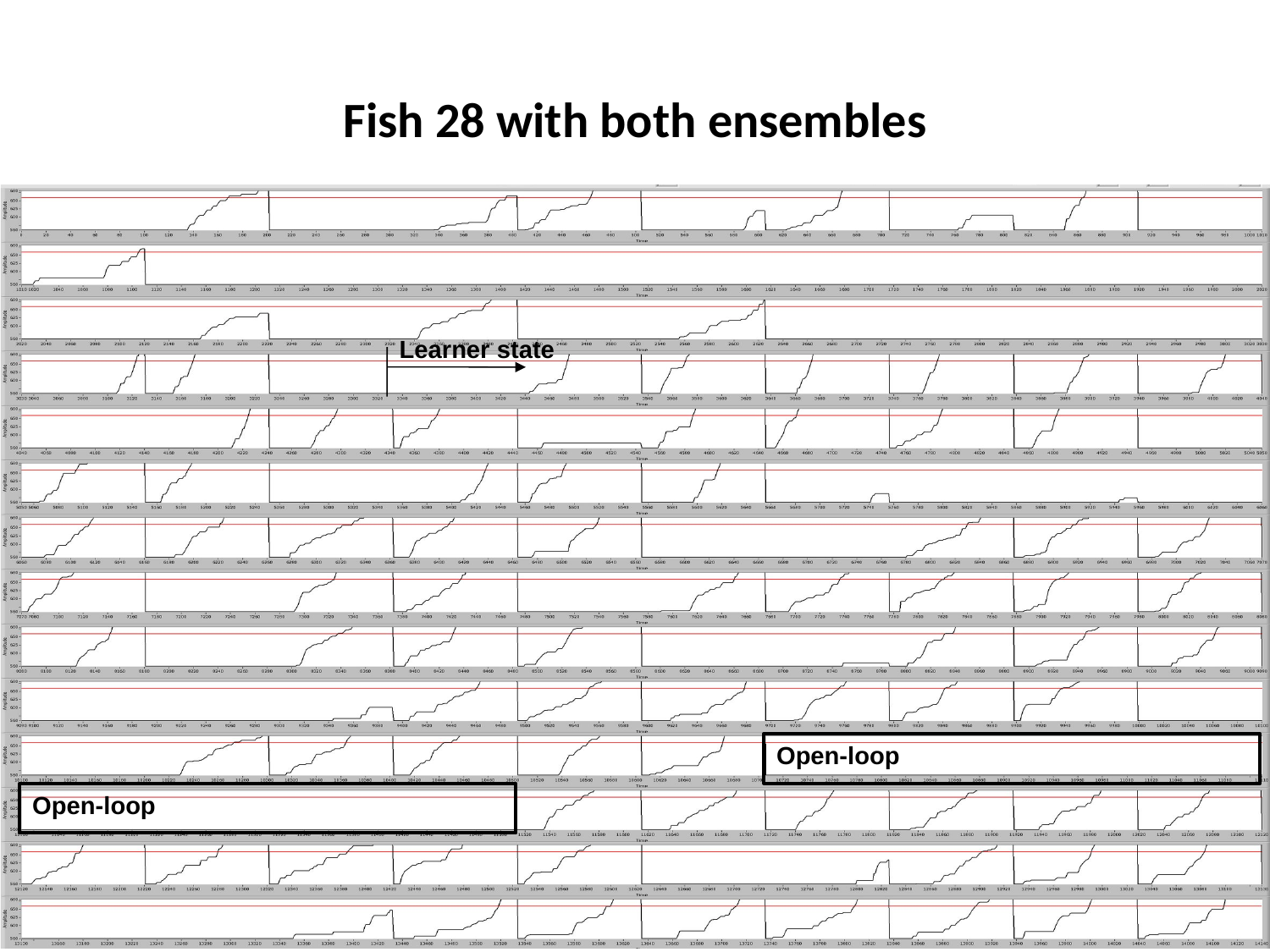

# Fish 28 with both ensembles
Learner state
Open-loop
Open-loop

## Slide 7
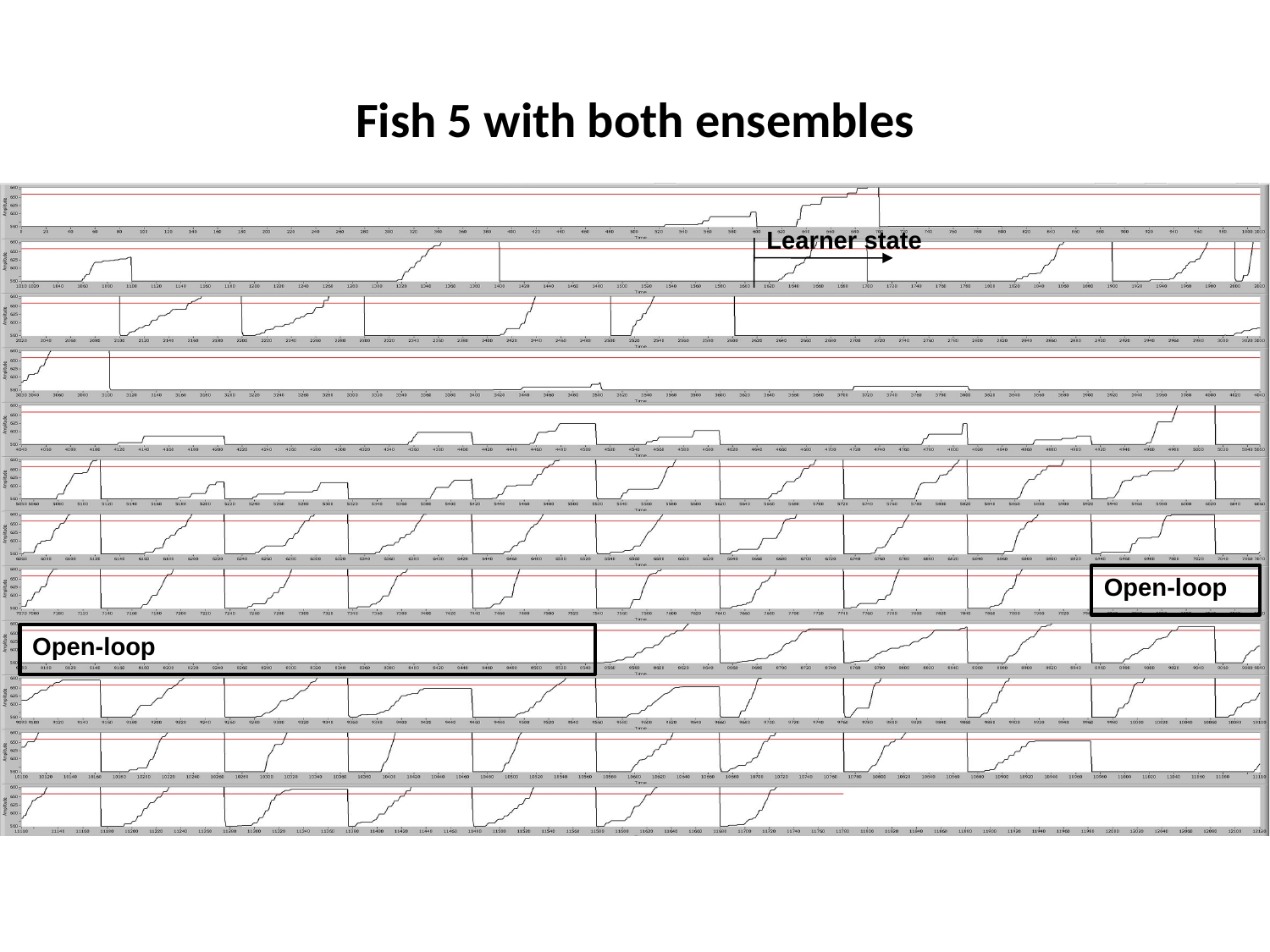

# Fish 5 with both ensembles
Learner state
Open-loop
Open-loop

## Slide 8
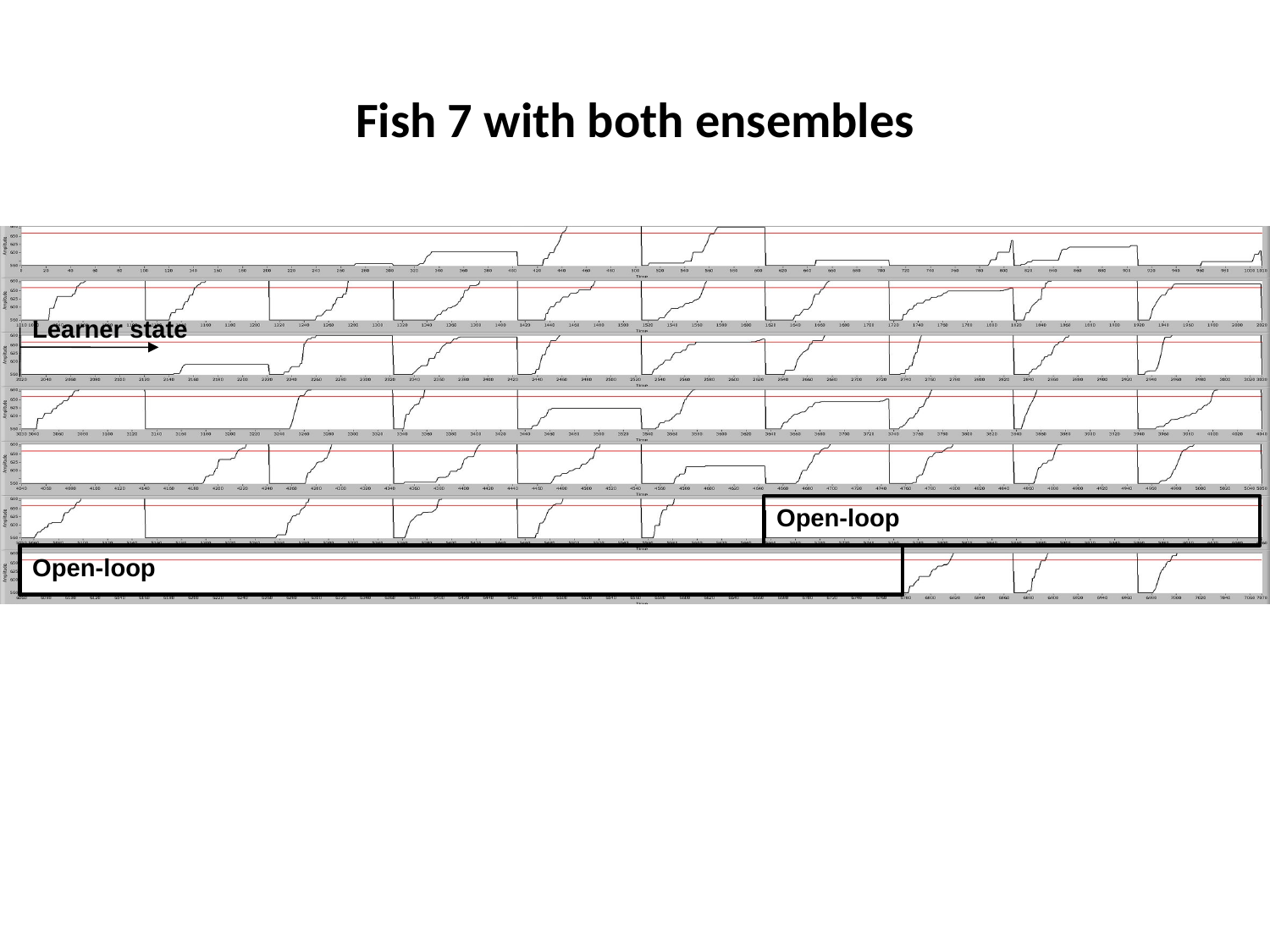

# Fish 7 with both ensembles
Learner state
Open-loop
Open-loop

## Slide 9
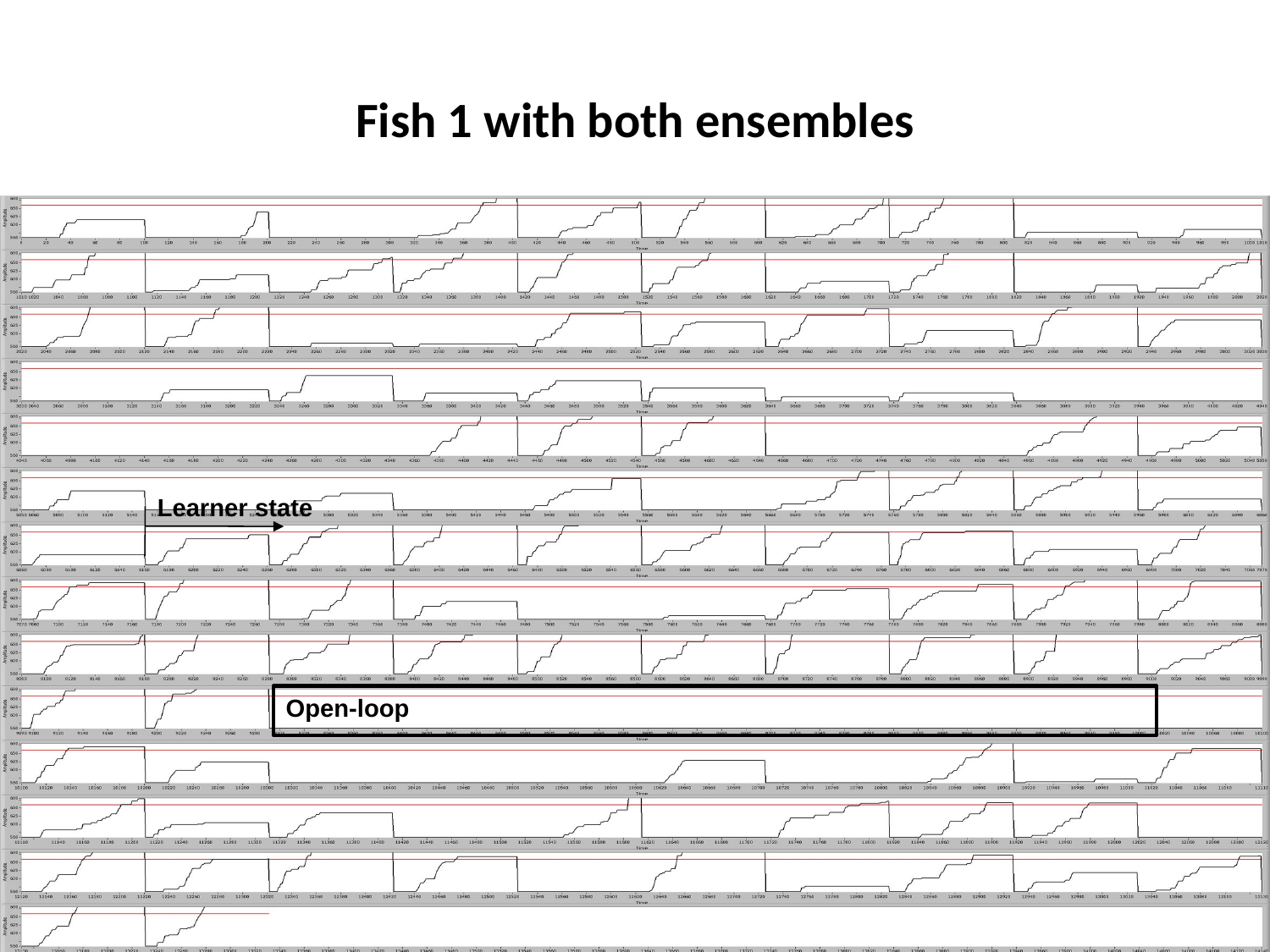

# Fish 1 with both ensembles
Learner state
Open-loop

## Slide 10
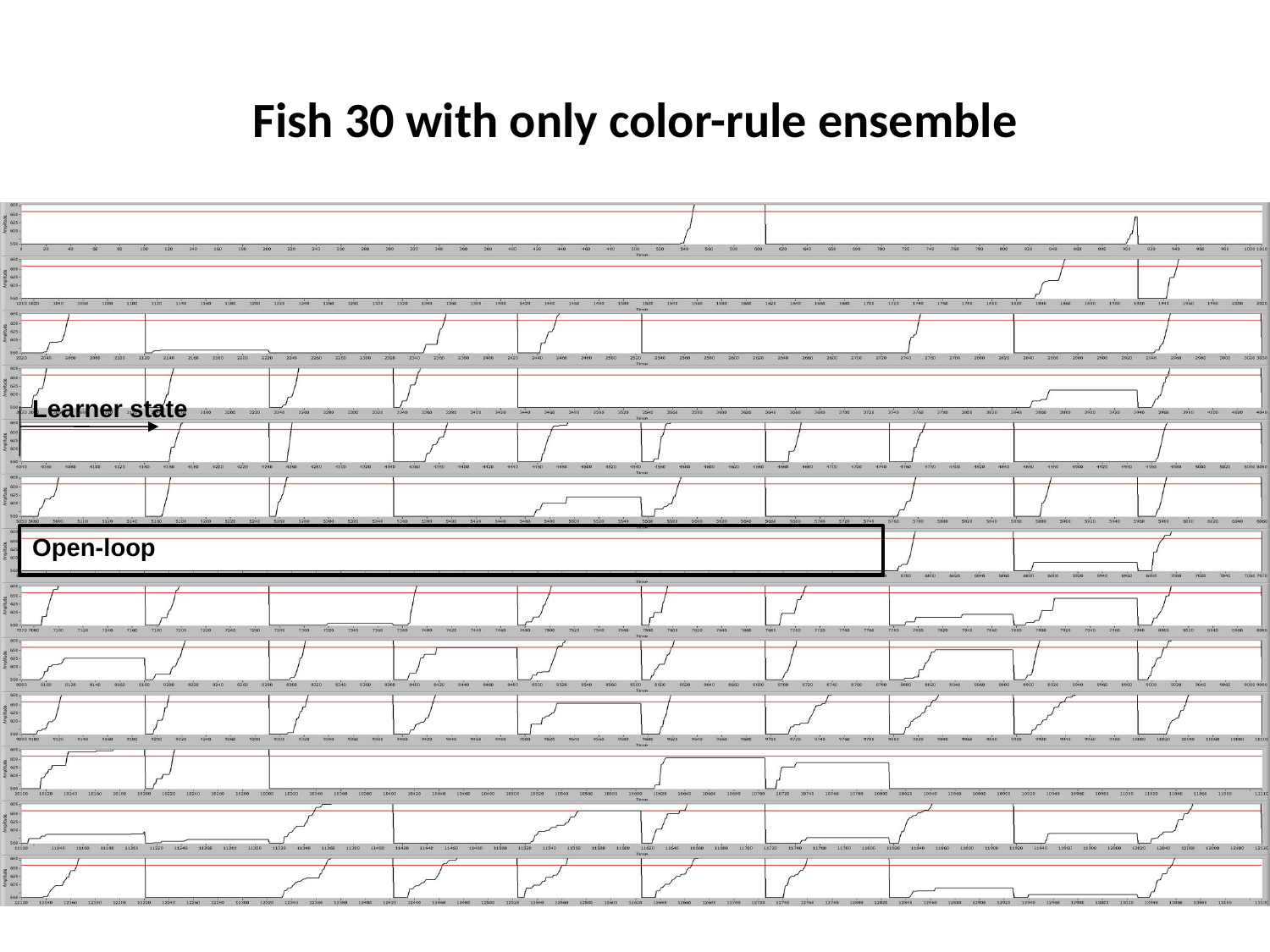

# Fish 30 with only color-rule ensemble
Learner state
Open-loop

## Slide 11
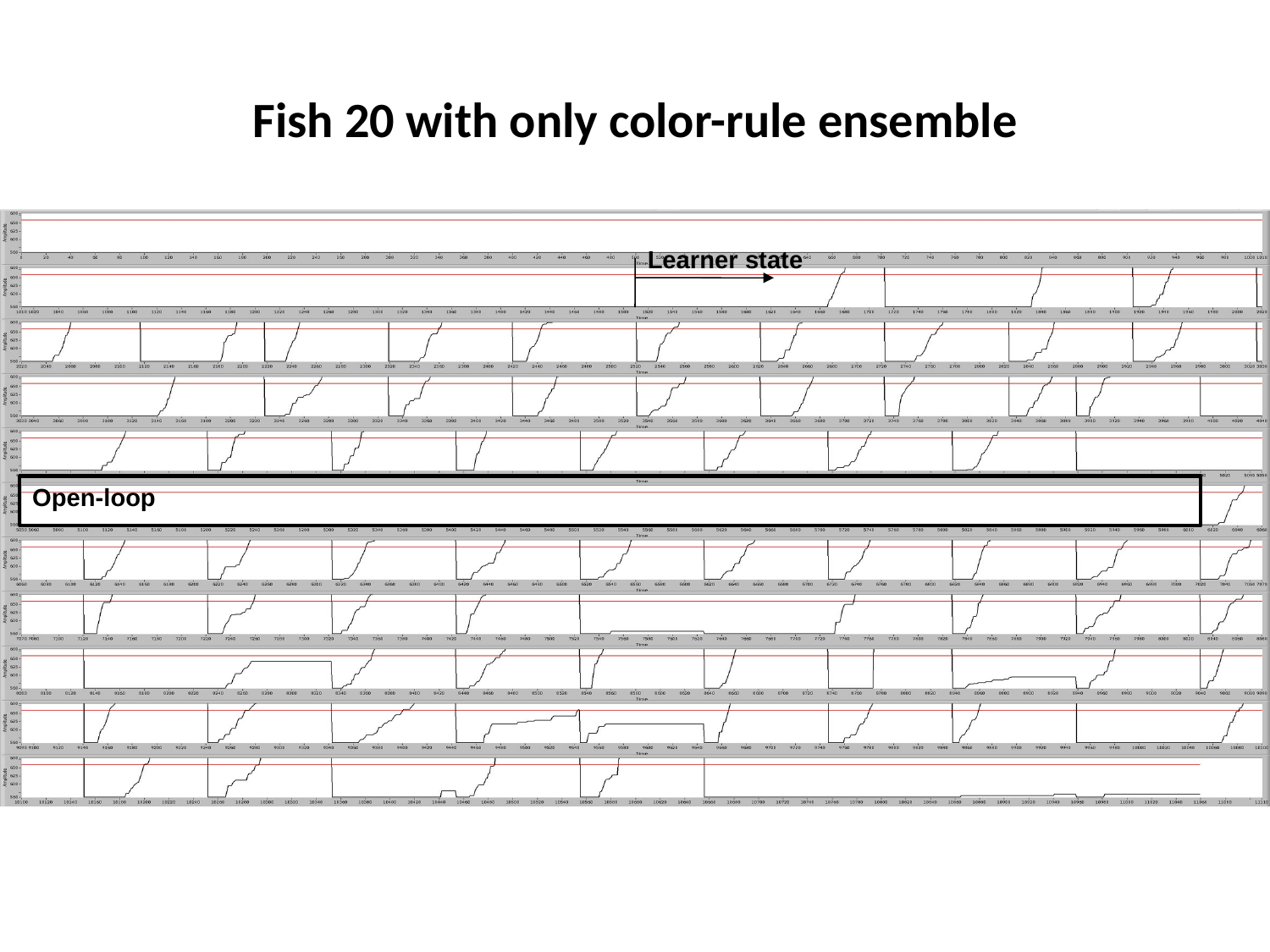

# Fish 20 with only color-rule ensemble
Learner state
Open-loop

## Slide 12
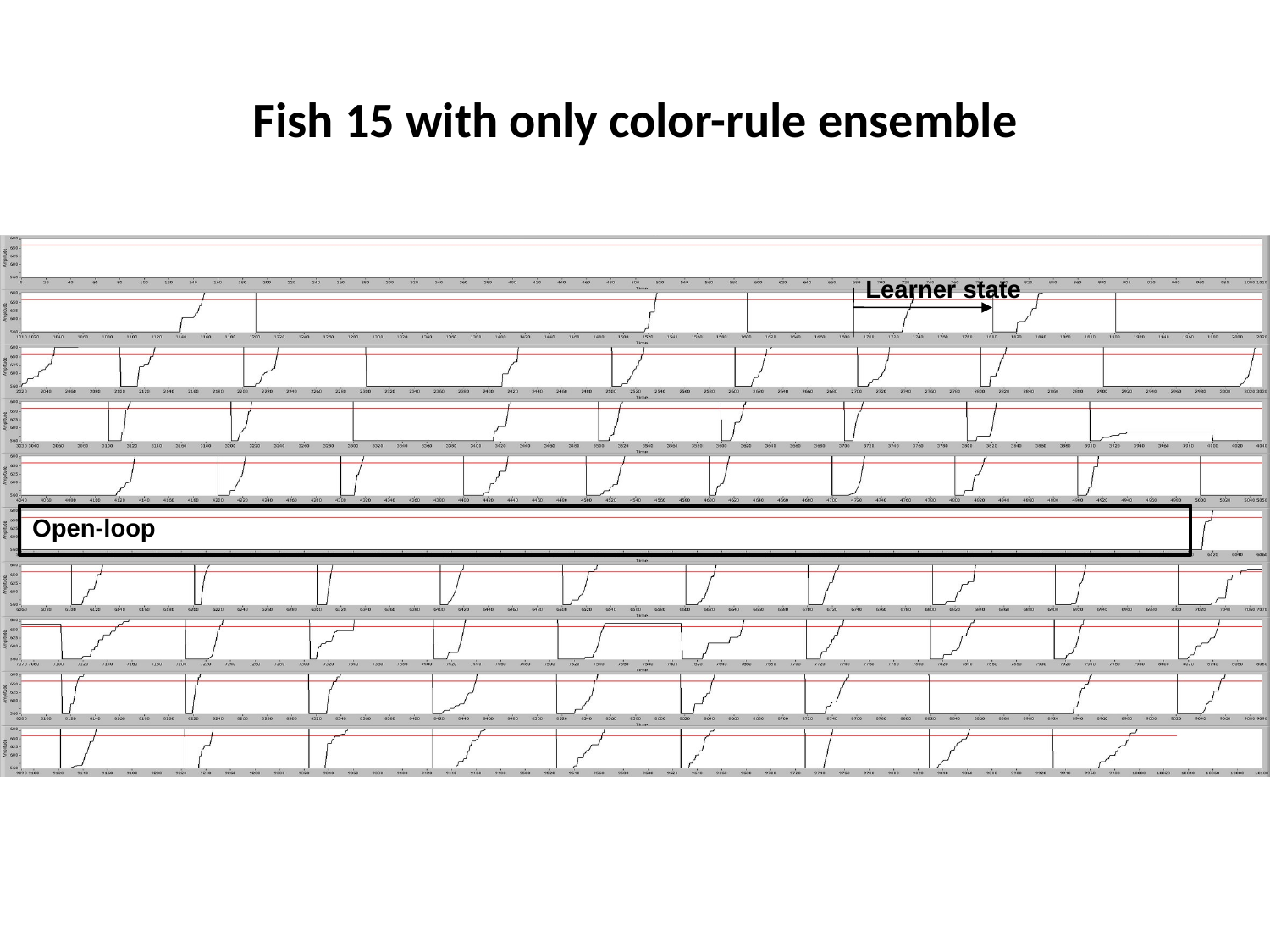

# Fish 15 with only color-rule ensemble
Learner state
Open-loop

## Slide 13
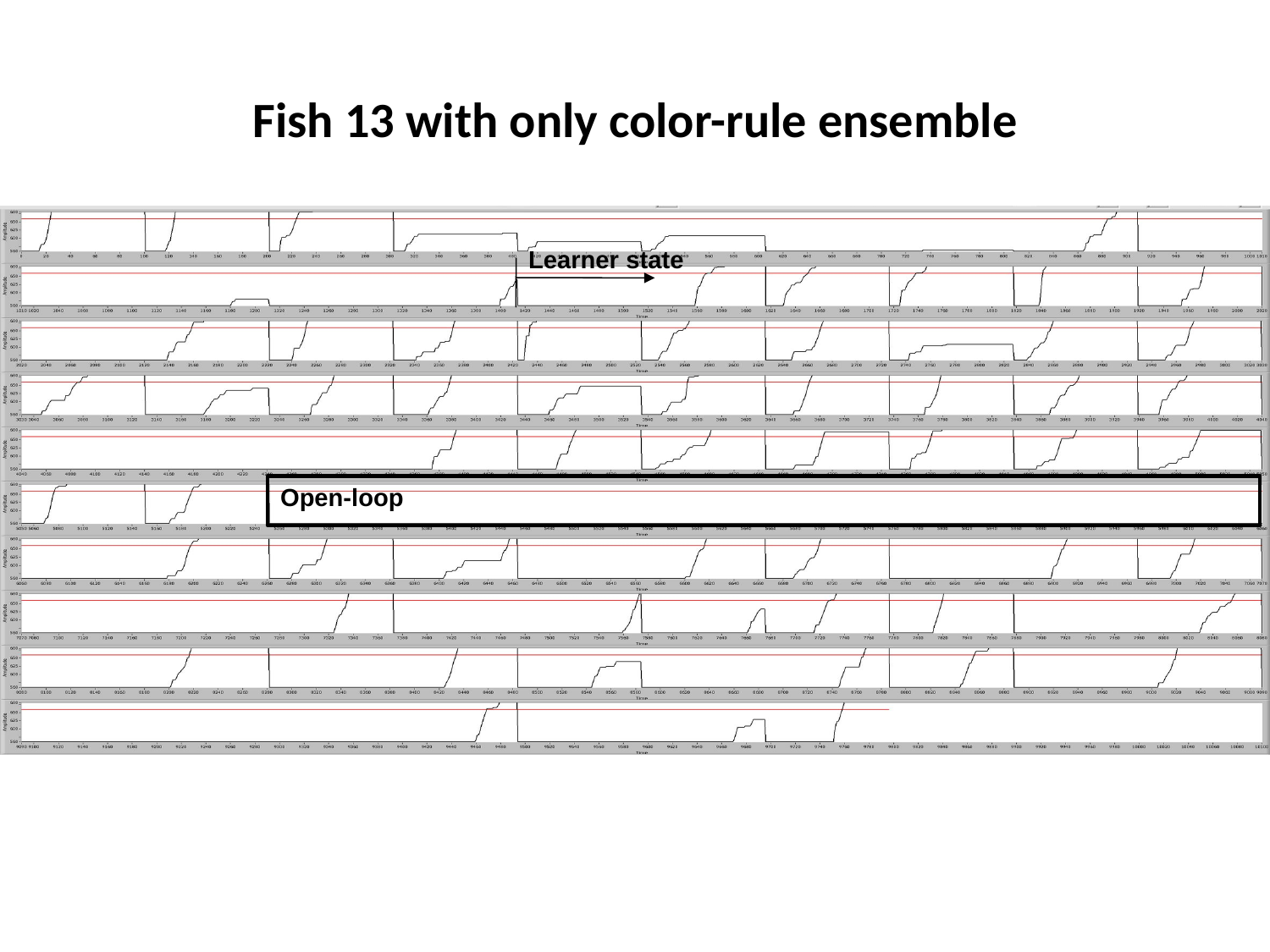

# Fish 13 with only color-rule ensemble
Learner state
Open-loop

## Slide 14
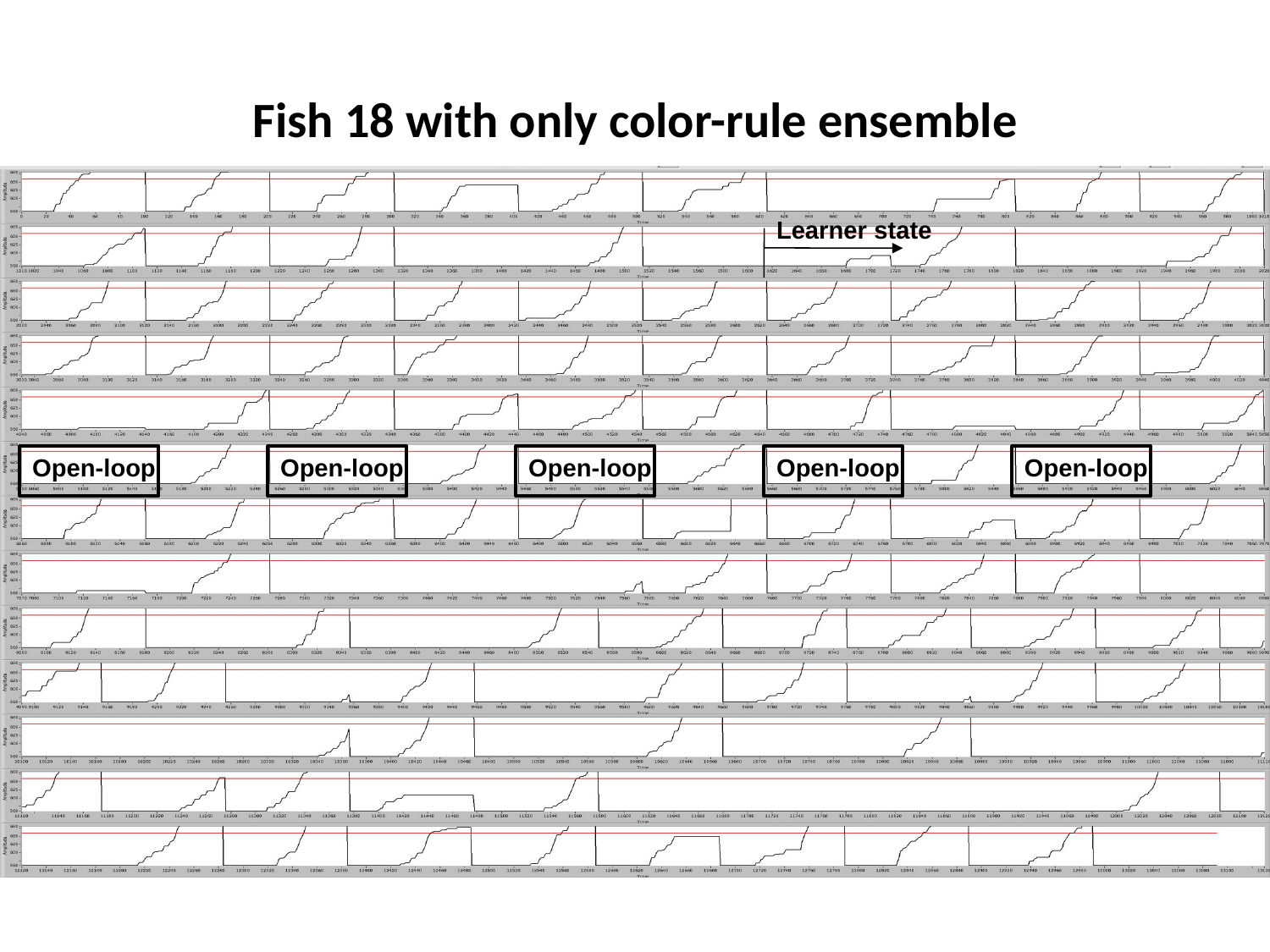

# Fish 18 with only color-rule ensemble
Learner state
Open-loop
Open-loop
Open-loop
Open-loop
Open-loop

## Slide 15
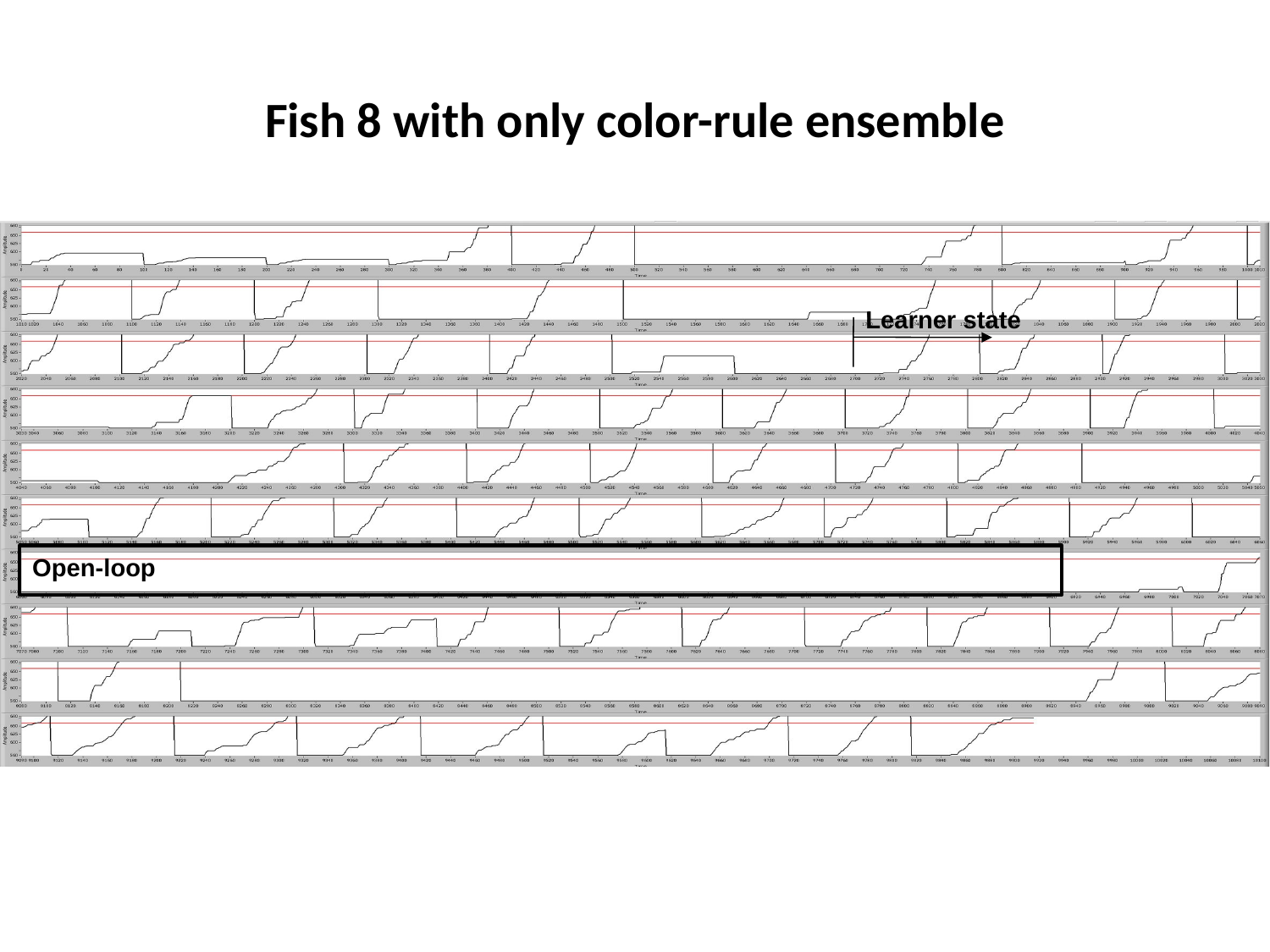

# Fish 8 with only color-rule ensemble
Learner state
Open-loop

## Slide 16
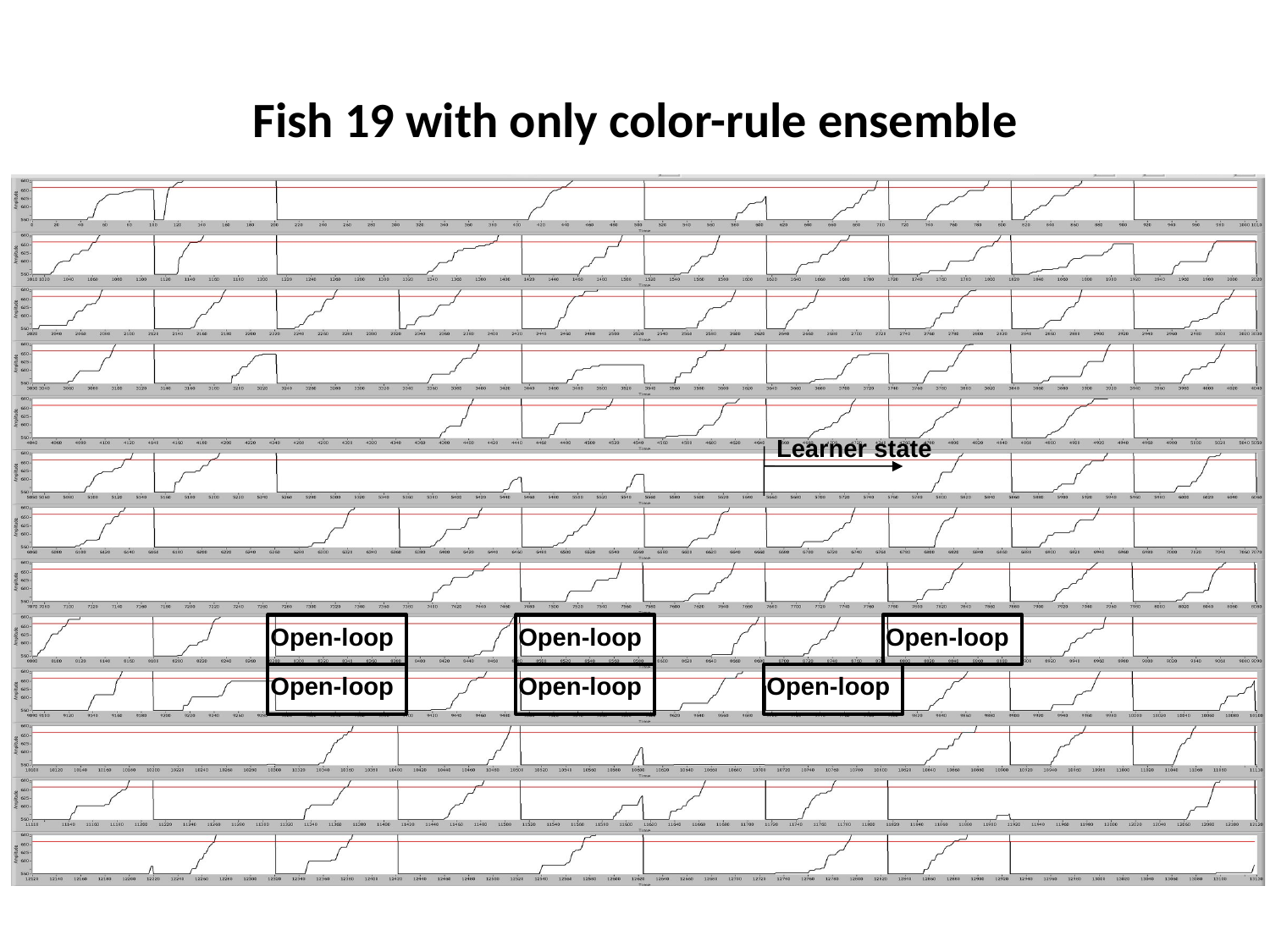

# Fish 19 with only color-rule ensemble
Learner state
Open-loop
Open-loop
Open-loop
Open-loop
Open-loop
Open-loop

## Slide 17
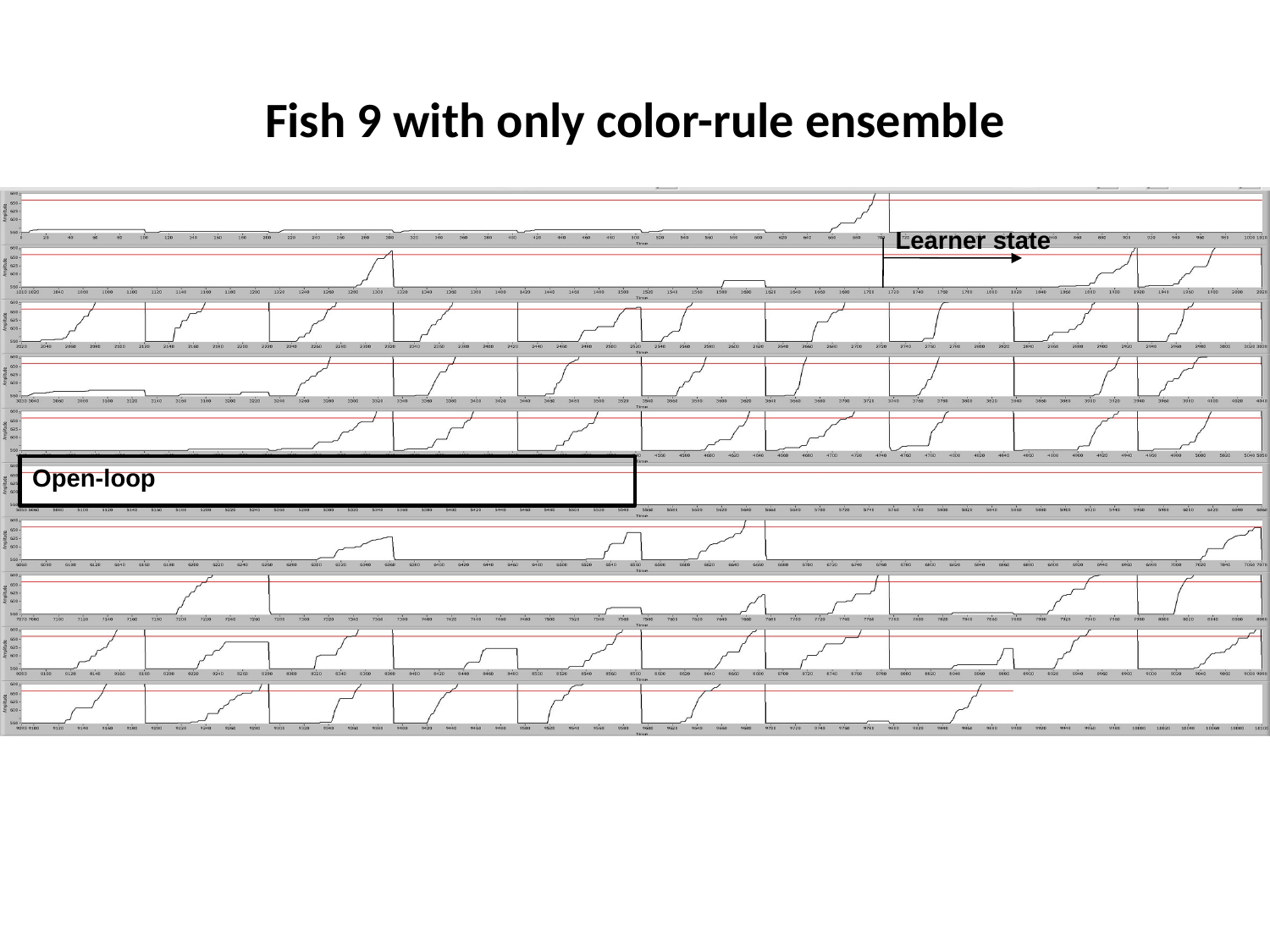

# Fish 9 with only color-rule ensemble
Learner state
Open-loop

## Slide 18
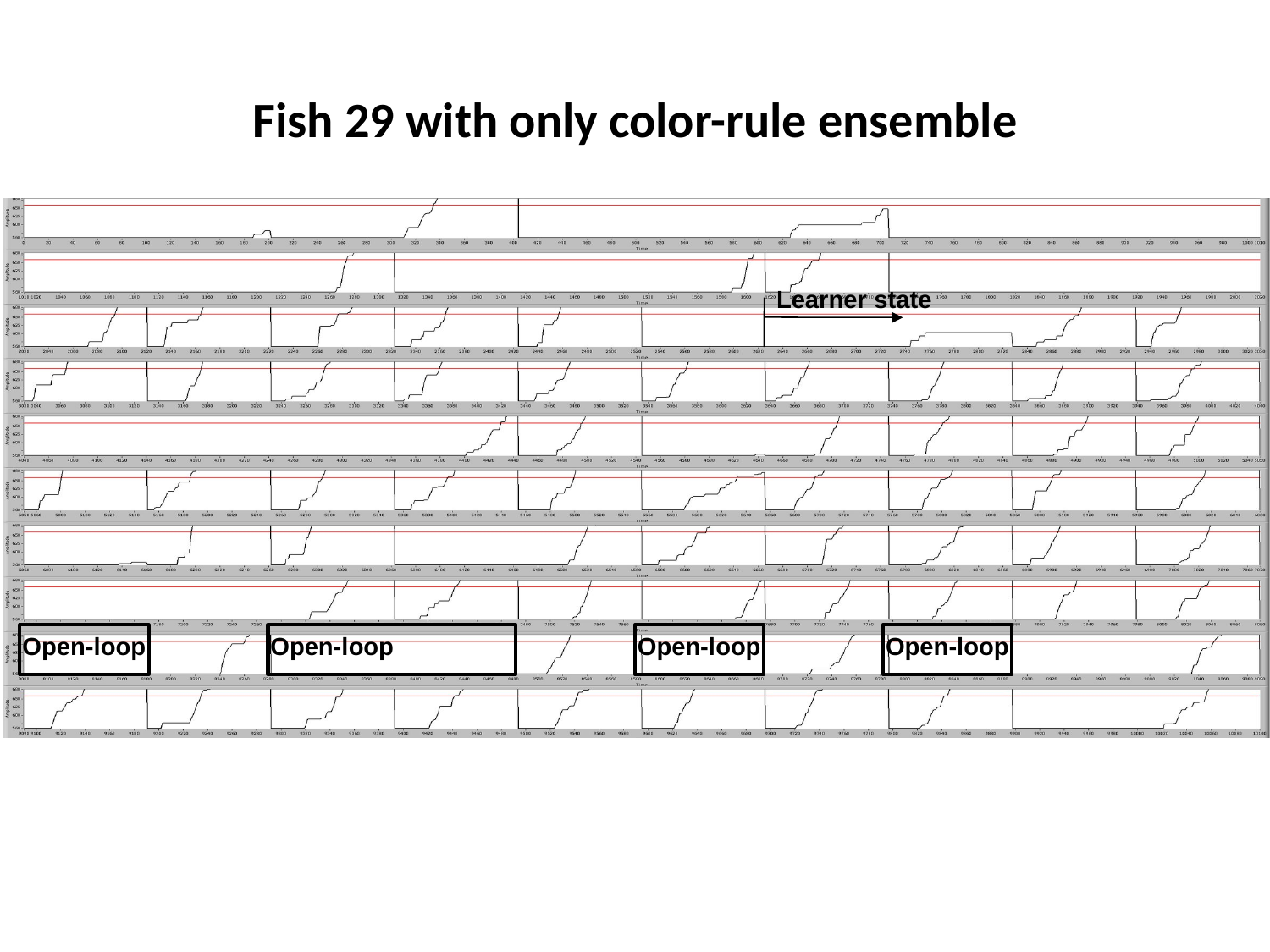

# Fish 29 with only color-rule ensemble
Learner state
Open-loop
Open-loop
Open-loop
Open-loop

## Slide 19
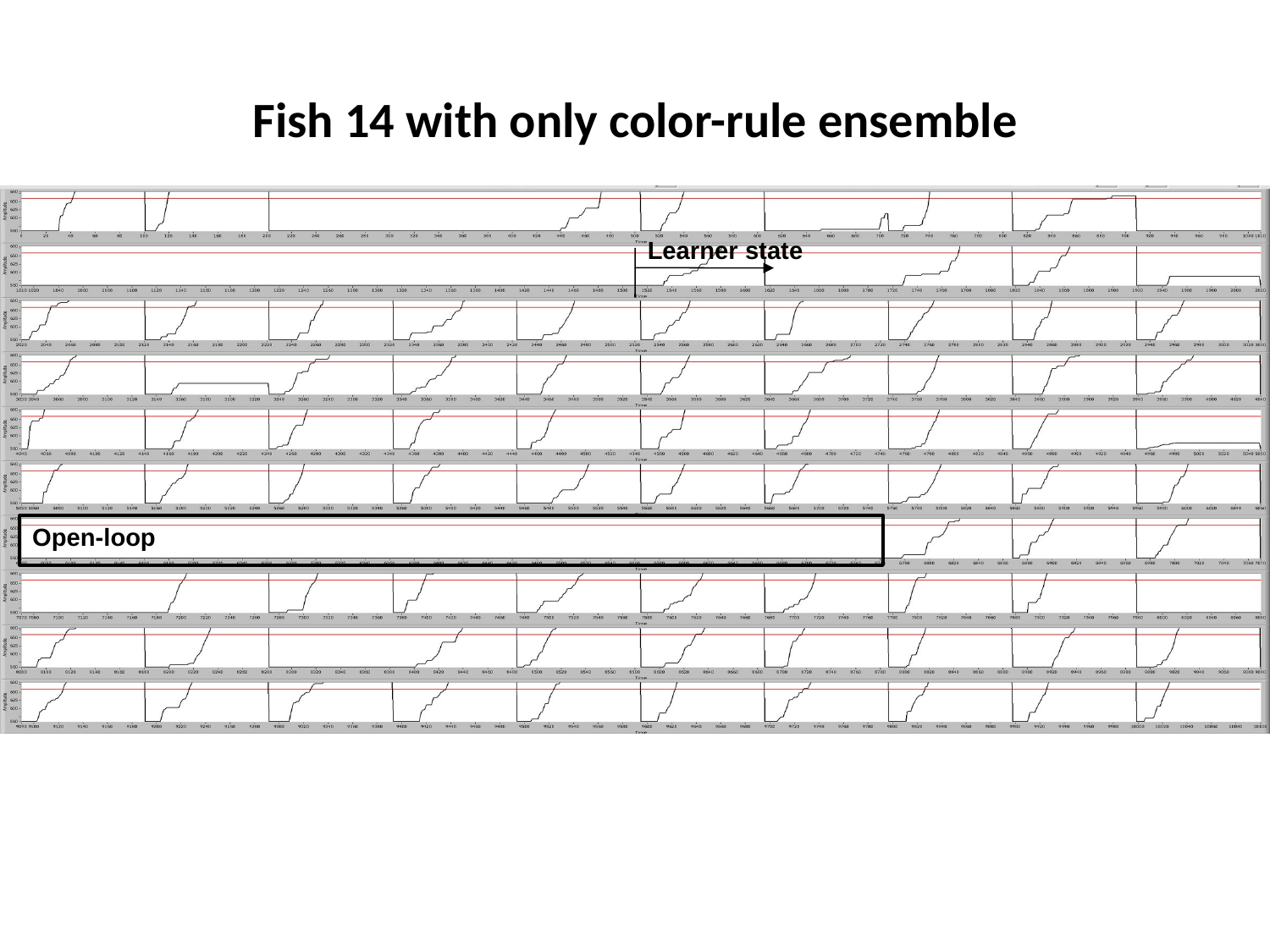

# Fish 14 with only color-rule ensemble
Learner state
Open-loop

## Slide 20
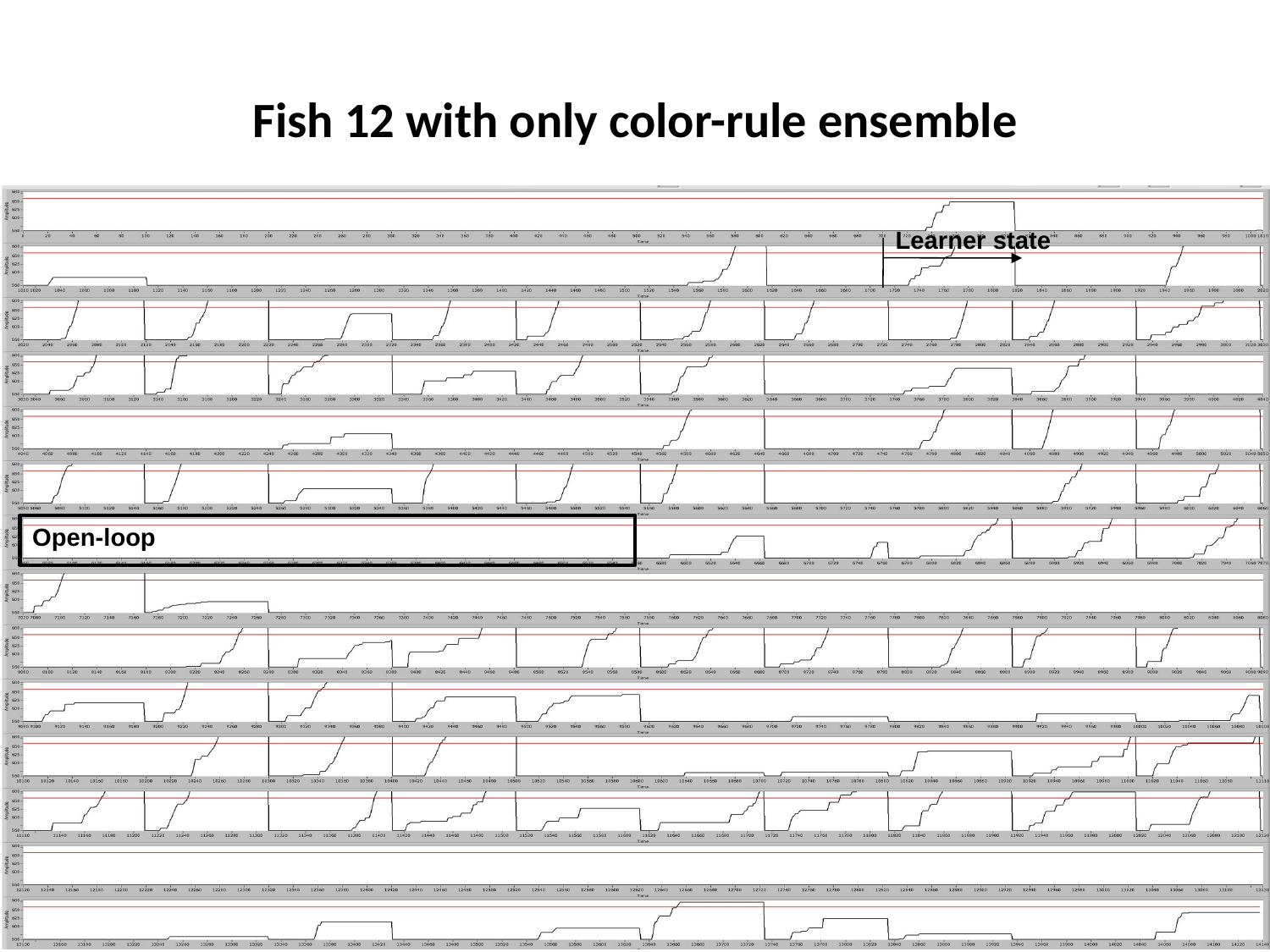

# Fish 12 with only color-rule ensemble
Learner state
Open-loop

## Slide 21
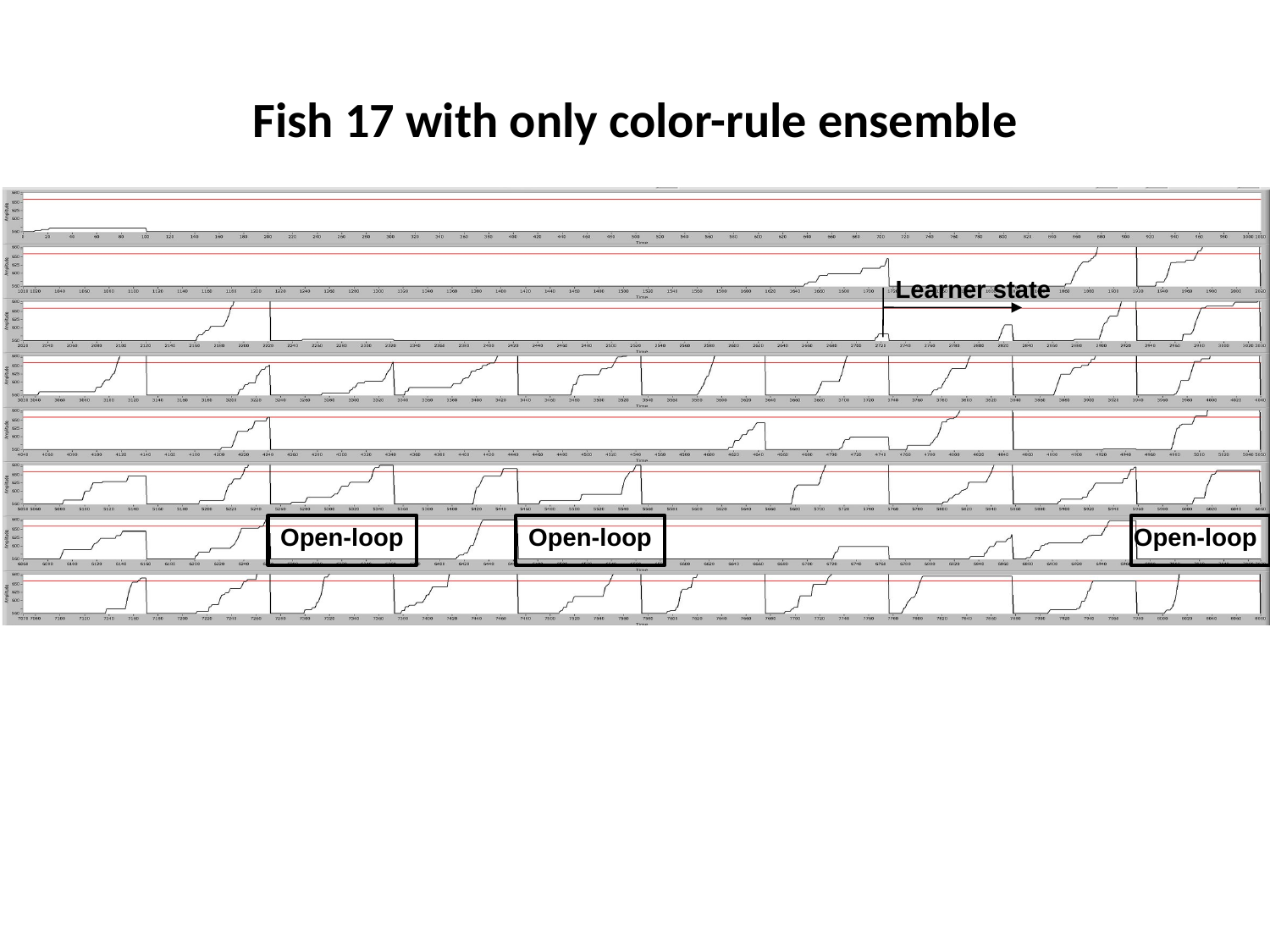

# Fish 17 with only color-rule ensemble
Learner state
Open-loop
Open-loop
Open-loop

## Slide 22
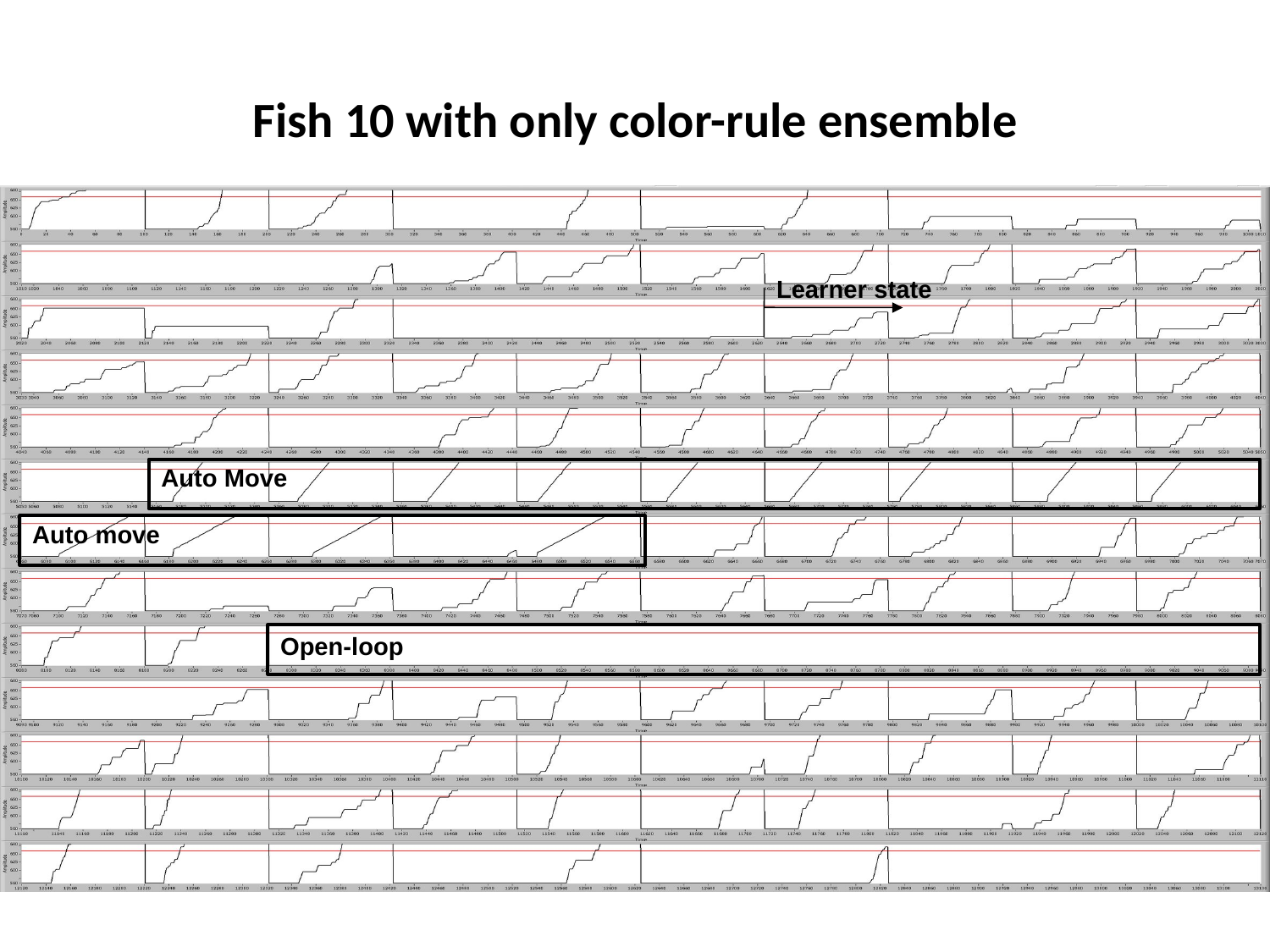

# Fish 10 with only color-rule ensemble
Learner state
Auto Move
Auto move
Open-loop

## Slide 23
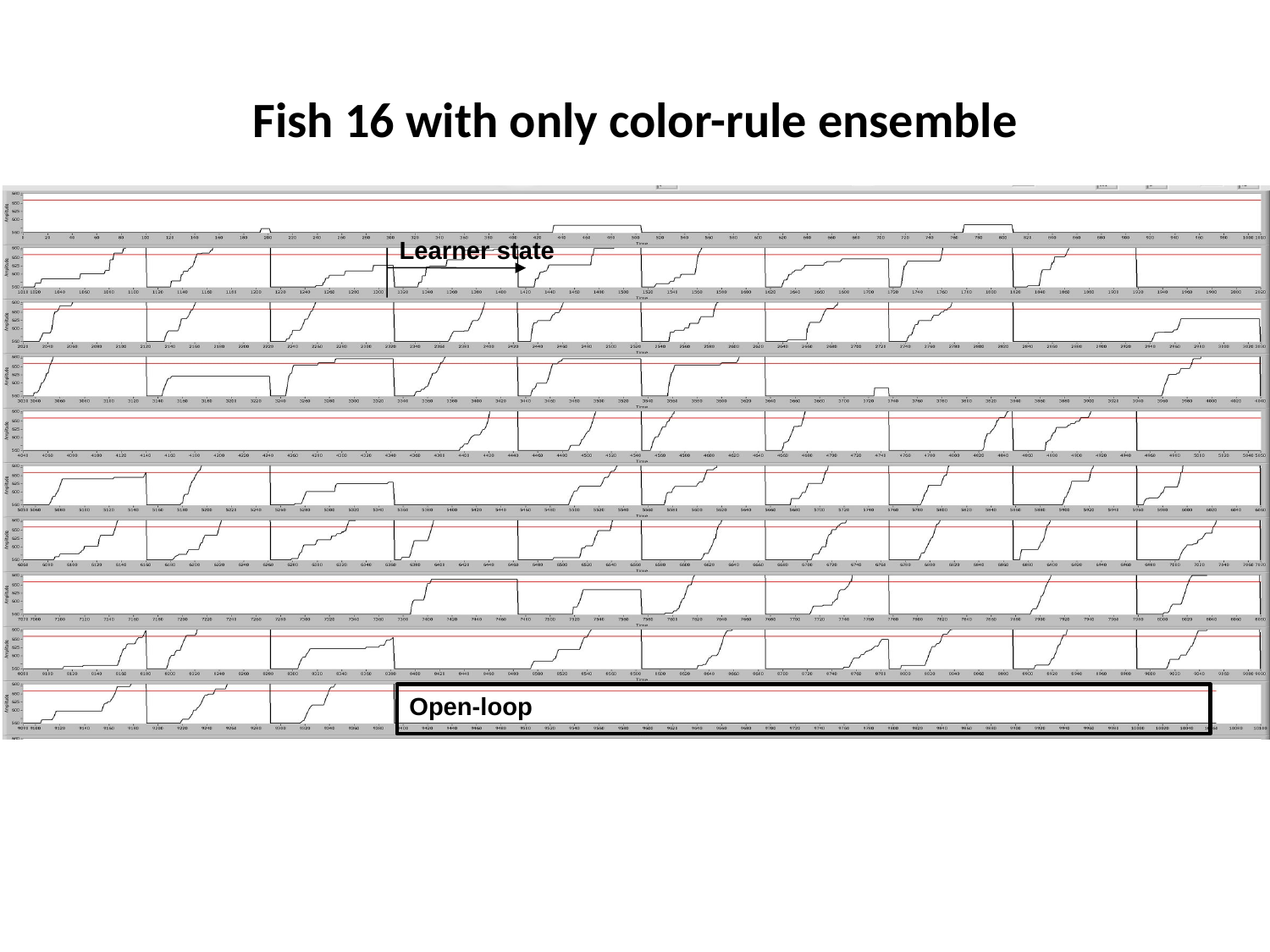

# Fish 16 with only color-rule ensemble
Learner state
Open-loop

## Slide 24
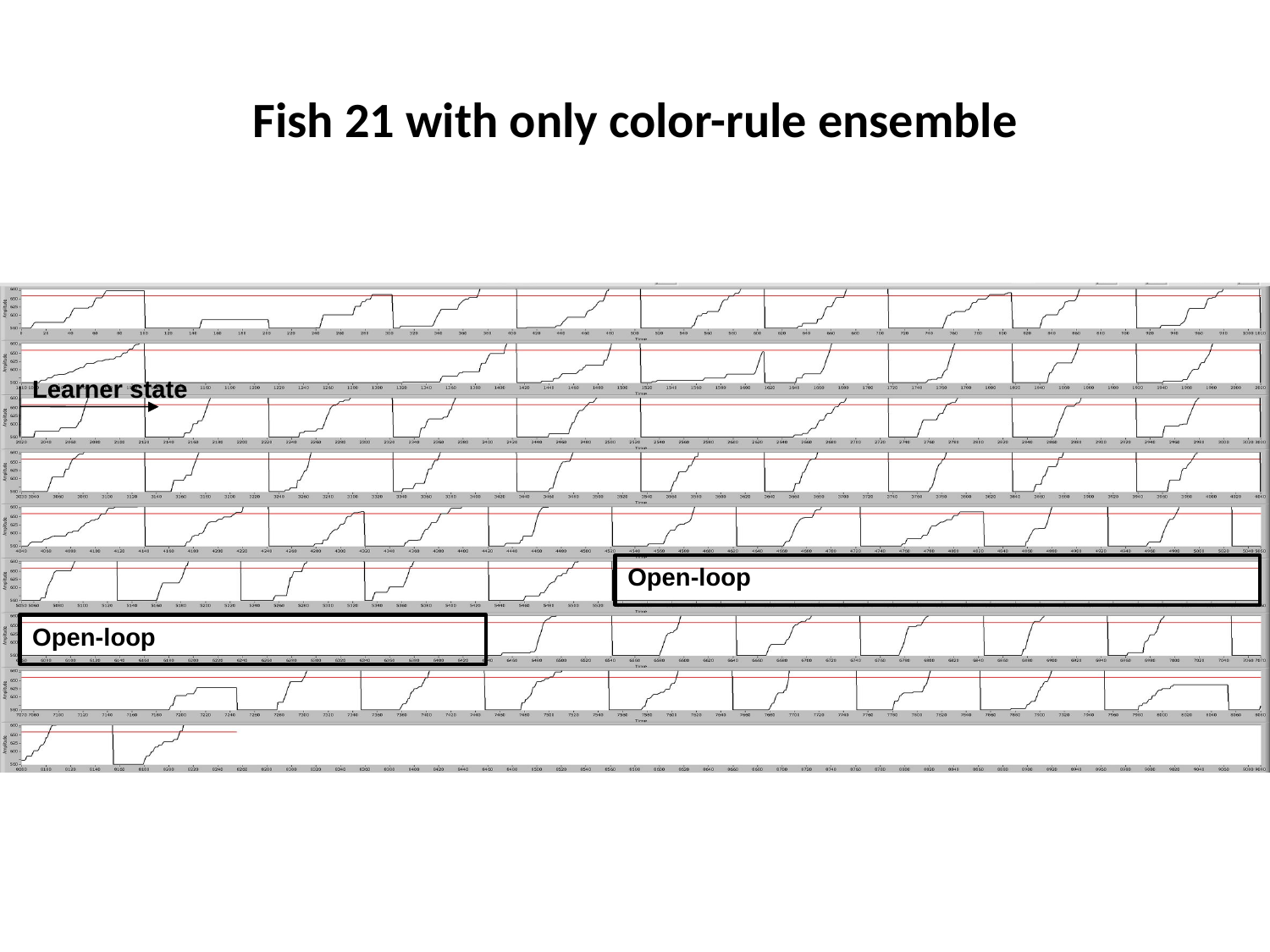

# Fish 21 with only color-rule ensemble
Learner state
Open-loop
Open-loop

## Slide 25
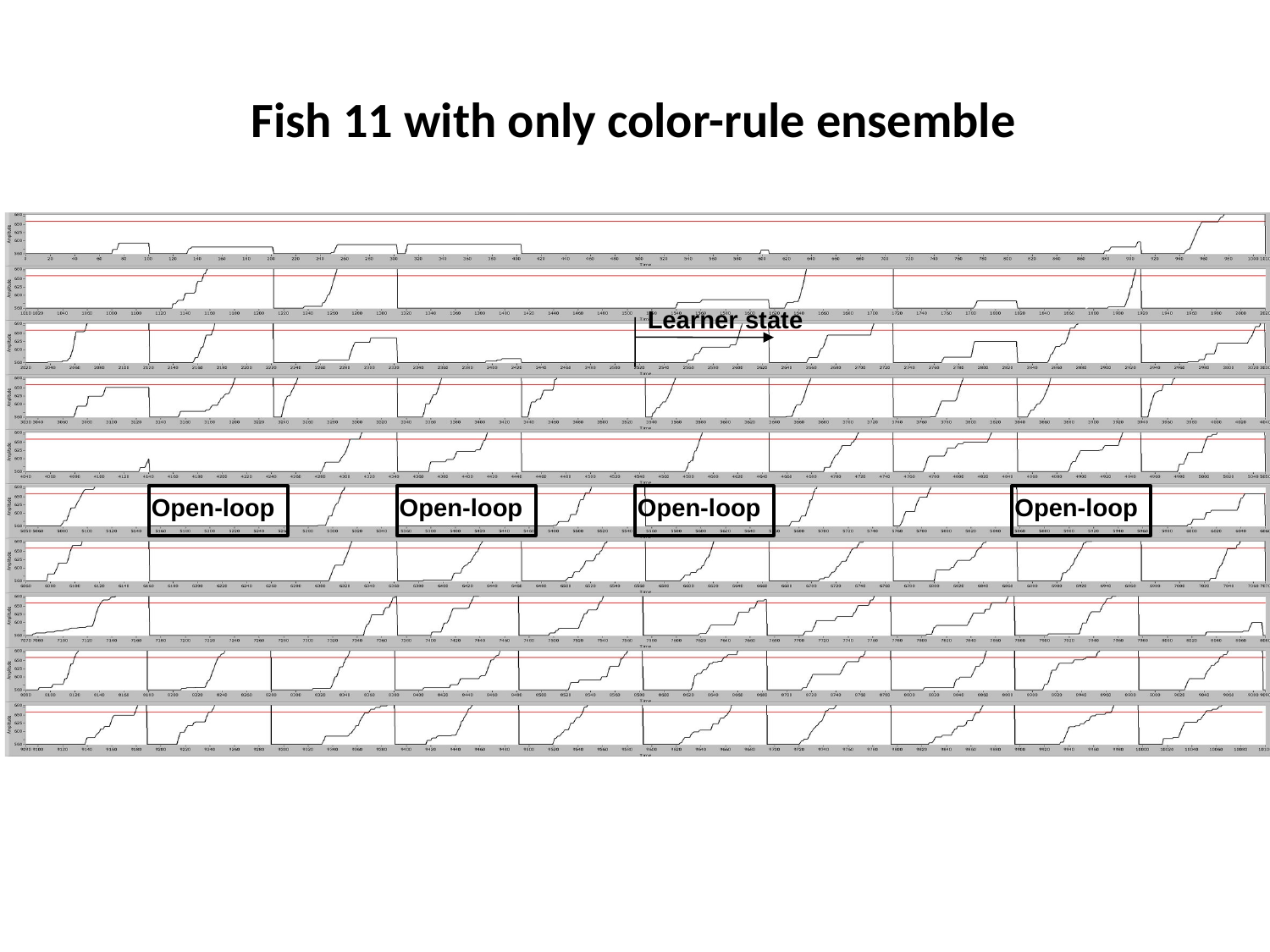

# Fish 11 with only color-rule ensemble
Learner state
Open-loop
Open-loop
Open-loop
Open-loop
