# Supplementary material for: Zebrafish capable of generating future state prediction error show improved active avoidance behavior in virtual reality
Source: Nat Commun. 2021 Sep 29;12:5712. doi: 10.1038/s41467-021-26010-7 (PMC8481257; doi:10.1038/s41467-021-26010-7)
Supplement: Supplementary file 8 — Reporting summary. [file 41467_2021_26010_MOESM8_ESM.pdf]

nature research

Corresponding author(s): Hitoshi Okamoto

Last updated by author(s): Aug 5, 2021

Reporting Summary

Nature Research wishes to improve the reproducibility of the work that we publish. This form provides structure for consistency and transparency in reporting. For further information on Nature Research policies, see our Editorial Policies and the Editorial Policy Checklist.

Statistics

For all statistical analyses, confirm that the following items are present in the figure legend, table legend, main text, or Methods section.

n/a	Confirmed	
☐	☒	The exact sample size (n) for each experimental group/condition, given as a discrete number and unit of measurement
☐	☒	A statement on whether measurements were taken from distinct samples or whether the same sample was measured repeatedly
☐	☒	The statistical test(s) used AND whether they are one- or two-sided<br>*Only common tests should be described solely by name; describe more complex techniques in the Methods section.*
☐	☒	A description of all covariates tested
☐	☒	A description of any assumptions or corrections, such as tests of normality and adjustment for multiple comparisons
☐	☒	A full description of the statistical parameters including central tendency (e.g. means) or other basic estimates (e.g. regression coefficient) AND variation (e.g. standard deviation) or associated estimates of uncertainty (e.g. confidence intervals)
☐	☒	For null hypothesis testing, the test statistic (e.g. F, t, r) with confidence intervals, effect sizes, degrees of freedom and P value noted<br>*Give P values as exact values whenever suitable.*
☒	☐	For Bayesian analysis, information on the choice of priors and Markov chain Monte Carlo settings
☒	☐	For hierarchical and complex designs, identification of the appropriate level for tests and full reporting of outcomes
☐	☒	Estimates of effect sizes (e.g. Cohen's d, Pearson's r), indicating how they were calculated

Our web collection on statistics for biologists contains articles on many of the points above.

Software and code

Policy information about availability of computer code

Data collection	OMEGA SPACE(v3.3), LabVIEW(v12.0.1f5), Matlab(R2016b)
Data analysis	ImageJ (v1.50i and 1.51p), LabVIEW(v12.0.1f5), Matlab(R2016b), Excel (Microsoft Office 2007)

For manuscripts utilizing custom algorithms or software that are central to the research but not yet described in published literature, software must be made available to editors and reviewers. We strongly encourage code deposition in a community repository (e.g. GitHub). See the Nature Research guidelines for submitting code & software for further information.

Data

Policy information about availability of data

All manuscripts must include a data availability statement. This statement should provide the following information, where applicable:

- Accession codes, unique identifiers, or web links for publicly available datasets
- A list of figures that have associated raw data
- A description of any restrictions on data availability

The data mainly mentioned in this study (fish 1,2,3) are available in the repository (). Remaining data are available from the corresponding author upon request.

Field-specific reporting

Life sciences study design

All studies must disclose on these points even when the disclosure is negative.

Sample size	No methods were used to predetermine sample sizes. In the main results (Fig. 7c), the effect size of t-test is the range of large and very large.<br>Fish group which experienced only original rule with 3-plane imaging: tested fish, 129; learner fish, 33; non learner fish, 96. All learner fish also experienced the open-loop condition.<br>Fish which experienced the original and reversed rules: tested fish, 52; fish which learned both original and reversed rules, 7; fish which learned the original rule but did not learn the reversed rules, 18.<br>Fish which experienced only the original rule with 6-plane imaging: tested fish, 24; learner fish, 4; non learner fish, 20. All learner fish experienced the open-loop condition.
Data exclusions	The exclusion criteria based on the behavioral learning were pre-established. If fish did not show behavioral learning in each rule, the data were excluded from the further analysis. In addition, if the imaging data exhibited large image displacement during the imaging period, the data were excluded from the further analysis.<br>Fish group which experienced only original rule with 3-plane imaging: the data of non learner fish and one leaner fish (because of the image displacement) were excluded.<br>Fish group which experienced original and reversed rules: the data of fish which did not learn either rules and two fish which learned both rules (because of the image displacement) were excluded.<br>Fish group which experienced only original rule with 6-plane imaging: the data of non-learner fish were excluded.
Replication	The analysis results were replicated in both 3-plane and 6-plane imaging experiments, which were performed independently. Each animal was tested independently. The number of fish for each experiment is reported for each experiment in the manuscript. The means and standard error are also reported.
Randomization	For each independent experiment, fish were randomly selected. No fish was used twice in our experiments.
Blinding	Blinding was not relevant to this study because, in this study, we only used one type of fish and judgment of learner or non-learner could be automatically determined by the behavior of fish which was not under the control of the experimenter.

Reporting for specific materials, systems and methods

We require information from authors about some types of materials, experimental systems and methods used in many studies. Here, indicate whether each material, system or method listed is relevant to your study. If you are not sure if a list item applies to your research, read the appropriate section before selecting a response.

Materials & experimental systems

n/a	Involved in the study
☒	☐ Antibodies
☒	☐ Eukaryotic cell lines
☒	☐ Palaeontology and archaeology
☐	☒ Animals and other organisms
☒	☐ Human research participants
☒	☐ Clinical data
☒	☐ Dual use research of concern

Methods

n/a	Involved in the study
☒	☐ ChIP-seq
☒	☐ Flow cytometry
☒	☐ MRI-based neuroimaging

Animals and other organisms

Policy information about studies involving animals; ARRIVE guidelines recommended for reporting animal research

Laboratory animals	In this study, we used TgBAC(camk2a:GAL4VP16)rw0154a; TgBAC(vglut2a:Gal4); Tg(UAS:G-CaMP7) rw0155 zebrafish aged >6 months in the nacre or casper background. Both male and female fish were used for all experiments (Supplementary Table 1 and 2).
Wild animals	We did not use wild animals in this study.
Field-collected samples	We did not use animals collected from field in this study.
Ethics oversight	All surgical and experimental procedures were reviewed and approved by the Animal Care and Use Committees of the RIKEN Center for Brain Science.

Note that full information on the approval of the study protocol must also be provided in the manuscript.
